# Social inference brain networks in autistic adults during movie-viewing: functional specialization and heterogeneity

**DOI:** 10.1186/s13229-025-00669-x

**Published:** 2025-08-22

**Authors:** Jasmin M. Turner, Lisa Byrge, Hilary Richardson, Paola Galdi, Daniel P. Kennedy, Dorit Kliemann

**Affiliations:** 1 Department of Psychological and Brain Sciences, The University of Iowa, 340 Iowa Avenue, Iowa City, IA 52242 USA; 2 Department of Psychological and Brain Sciences, University of North Florida, 1 UNF Drive, Jacksonville, FL 32224 USA; 3 Department of Psychology, University of Edinburgh, 7 George Square, Edinburgh, EH8 9JZ UK; 4 School of Informatics, University of Edinburgh, 10 Crichton Str, EH89AB, Edinburgh, Scotland; 5 Department of Psychological and Brain Sciences, Indiana University, 1101 E. 10th St, Bloomington, IN 47405-7007 USA; 6 Department of Psychological and Brain Sciences, Department of Psychiatry, and Iowa Neuroscience Institute, The University of Iowa, 340 Iowa Avenue, Iowa City, IA 52242 USA

**Keywords:** Autism, Theory of mind, Empathy, fMRI, Functional connectivity, Social cognition, Heterogeneity

## Abstract

**Background:**

Difficulty in social inferences is a core feature in autism spectrum disorders (ASD). On the behavioral level, it remains unclear whether reasoning about others’ *mental* states (Theory of Mind, ToM) and empathic responses to others’ *physical* states may be similarly or differentially affected in autism. On the neural level, these inferences typically engage distinct brain networks (ToM versus Pain networks), but their functional specialization remains not well understood in autism. This study aimed to investigate the functional specialization, heterogeneity, and brain-behavior relationships of the ToM and Pain networks in autistic compared to neurotypical (NT) participants. We hypothesized differential functional network specialization (i.e., functional connectivity), increased heterogeneity, and less typical network responses specifically in the ToM network, with relatively similar responses in the Pain network in ASD.

**Methods:**

Using functional magnetic resonance imaging (fMRI), we investigated neural responses in 107 adults (autistic: 34 (female = 11), NT: 73 (female = 23); matched for age, intellectual functioning, sex, motion) while they passively watched a short, animated movie including events that evoke reasoning about characters’ mental states and bodily sensations. Preregistered analyses included regression models to assess inter-region correlation of within- and across-network connectivity, inter-subject correlation to quantify similarity to the average neurotypical, as well as to within- and across-group timecourse responses, and brain-behavior relationships relevant for social inferences.

**Results:**

Functional specialization of ToM and Pain networks were overall intact, with distinct network responses in both groups. The autistic group showed differential ToM network responses and reduced similarity to the average typical response for both networks. Network responses were more idiosyncratic and heterogenous in the autistic group. Brain-behavior relationships differed between groups for ToM behavior only.

**Limitations:**

Effects between groups were overall small. Samples were acquired across two sites, yet the sample size restricts subgroup analyses that may further inform autistic heterogeneity and limits generalizability.

**Conclusions:**

We found weak evidence for greater differential responses in brain networks underlying ToM inferences than those involved in empathic responses in autism, consistent with a prior empathy imbalance hypothesis. We outline suggestions for replicating, generalizing and extending these results in future research.

**Supplementary Information:**

The online version contains supplementary material available at 10.1186/s13229-025-00669-x.

## Background

Identifying and understanding others’ internal states is an important skill underlying the ability to form and maintain successful interpersonal relationships. Individuals with a diagnosis of autism spectrum disorder (ASD) often face challenges in social cognition, marked by difficulties in comprehending others’ internal mental (i.e., cognitive and emotional) states [1]. Reasoning about others’ *mental* states (e.g., “what they think and emotionally feel”) and an empathic response to others *physical* states (“feeling what the other person is *bodily* feeling”, i.e., hunger, pain, fatigue) involve reasoning about different kinds of internal states, and may in fact be differentially affected in autism.

The ability to make inferences about others’ thoughts, beliefs and intentions (Theory of Mind, ToM [2–4]; sometimes also referred to as “mentalizing” [5, 6] is a crucial aspect in human social development [7, 8], and often delayed in autistic children [9–11]. In autistic adults, difficulty in ToM reasoning has been most commonly demonstrated in perspective-taking and mental state attribution tasks [12–17]. While much attention has been focused on the cognitive reasoning about others’ mental states in ASD, there is also a considerable, yet smaller, body of work investigating emotional contagion, considering physical sensations and bodily feelings. Specifically, the capacity to feel or emotionally resonate with another person [18–25] has received comparatively less attention in autism research. Autism had previously been described as “empathy disorder” [26]. Stereotypes and stigmatizing prejudices paint autistic people as “emotion-less” [27, 28] with impacts on individuals. Empathy (here: specifically emotional empathy; see [29–31] for discussions on emotional vs. cognitive empathy) may, however, be less affected, relatively intact or even enhanced in autistic people [32–35]. For example, prior studies reported typical or heightened emotional empathy when tasks involved emotional stimuli (e.g., photographs depicting people in emotionally charged scenarios, watching others in pain) or responding to more real-life emotional scenarios [35, 36]. For instance, studies have reported similar levels of emotional empathy in autistic and neurotypical participants when matched for intellectual functioning [30, 34]. Others have investigated specific aspects of emotional empathy and point to difficulty in appropriately modulating emotional responses and empathic concern [37].

On the level of brain function, previous research has identified two distinct networks of brain regions specialized in processing certain aspects of others’ internal states. Regions in the ToM network (sometimes referred to as the mentalizing network [5]) respond preferentially to internal mental states (e.g., beliefs, desires, intentions and emotions), and include the precuneus cortex (PC), medial prefrontal cortex (MPFC), bilateral temporoparietal junction regions (TPJ) [38–47]. Regions in the Pain Network (sometimes referred to as the “Pain Matrix” [48, 49]), respond preferentially to physical sensations and bodily feelings (e.g., pain, hunger, fatigue), and include the anterior middle cingulate cortex (AMCC), as well as bilateral medial frontal gyrus (MFG), anterior insula (AI), and secondary sensory cortex [24, 50–53].

Differentiation of cortical responses to representations of others’ mental and physical states is a key aspect of development in the brain regions sensitive to processing social information (the so-called “social brain” [2]). These two networks are uncorrelated in children as young as three years of age. As children get older, the brain activity within each network becomes more correlated with one another, and the two networks become increasingly specialized [54, 55]. Adults typically show a functional specialization across these two networks, with distinct activations in ToM and Pain networks in response to mental and bodily events, respectively. In contrast, younger children exhibit more cross-network activity. Here, the timecourse “maturity” (similarity, i.e., how correlated each child’s timecourse was to the average adult timecourse) was related to the extent to which the responses in ToM and Pain networks were anti-correlated. For example, movie scenes that evoke responses in the Pain network in adults can trigger ToM network activity, and vice versa, in 3-year-olds [54, 56]. Overall, these findings suggest that the development of functionally specialized brain regions for understanding other people’s thoughts, feelings and physical sensation involves increasingly specific responses across these two networks.

Similarly to the behavioral findings on internal state inferences in ASD described above, the focus has largely been on brain function related to challenges in understanding mental states, with less focus on the neural representation of others’ bodily feelings in autism. The functional specialization of these networks in autistic participants remains still not well understood, and brain imaging studies show inconsistent findings. Regarding the neural basis underlying difficulty in ToM reasoning, some studies report hypoactivation (i.e., reduced magnitude responses) of key regions in the ToM network, e.g., in the MPFC, left and right TPJ (lTPJ, rTPJ), and PC during mentalizing tasks [57–60]. Conversely, other studies reported hyperactivation (i.e., increased response magnitude) in the same regions [61, 62]. Other studies reported similar levels of brain activity compared to comparison groups across all regions in the ToM network [63–65]. With regards to empathic responses, prior studies on this topic have reported similarly inconsistent results in the Pain network in autistic as compared to neurotypical participants [32, 66, 67], possibly due to using different types of stimuli [68]. Taken together, findings to date suggest that autistic people may exhibit changes in mental state inferences, while ‘embodied’ aspects of empathy (including inferring bodily feelings / physical sensations) may be more similar (i.e., less affected) on both the behavioral and brain function level.

Few studies to date have investigated mental and bodily state inferences and related brain network specialization in the ToM and Pain networks in one experimental paradigm, and in the same participants. In fact, standard univariate analyses using traditionally highly controlled experiments often analyzed with general linear models may not be best suited to studying the complex social difficulties associated with internal state inferences in ASD [63, 69]. Such experiments may often lack ecological validity, given their reliance on stimuli that may not reflect the dynamic, context-dependent nature required for social information processing in real-world social interactions [70], including the integration of contextual information over time. As such, these studies may overlook diagnostically relevant autistic behaviors related to their everyday experiences outside the lab [63, 69, 71–73]. The use of dynamic, semi-naturalistic stimuli, such as movies, is an alternative approach that may be better suited to capturing individual differences, as well as subtle alterations in samples, such as ASD [74–77], while providing reliable results even across different acquisition sites [77]. Passive movie-watching paradigms are without direct, concurrent measurements of task performance; however, neural responses have been shown to nevertheless be relevant for social cognitive behavior outside of the scanner [54, 55, 74]. Additionally, they offer the advantage of minimizing explicit task instructions that may otherwise interfere with ecological and spontaneous social cognition [78, 79]. This may be particularly relevant for identifying more subtle difficulties in autistic adults with at least typical IQ and intact language abilities. Movie-watching paradigms are also particularly well suited for inter-regional and inter-subject correlation approaches, (IRC/ISC [75, 80]), which assess similarity of evoked responses within and across individuals and groups, and within and across functional brain networks. These data-driven approaches have been especially useful for measuring functional specialization of brain networks [81, 82], and for comparing brain responses in clinical populations to “normative” reference groups [83].

**Fig. 1:**
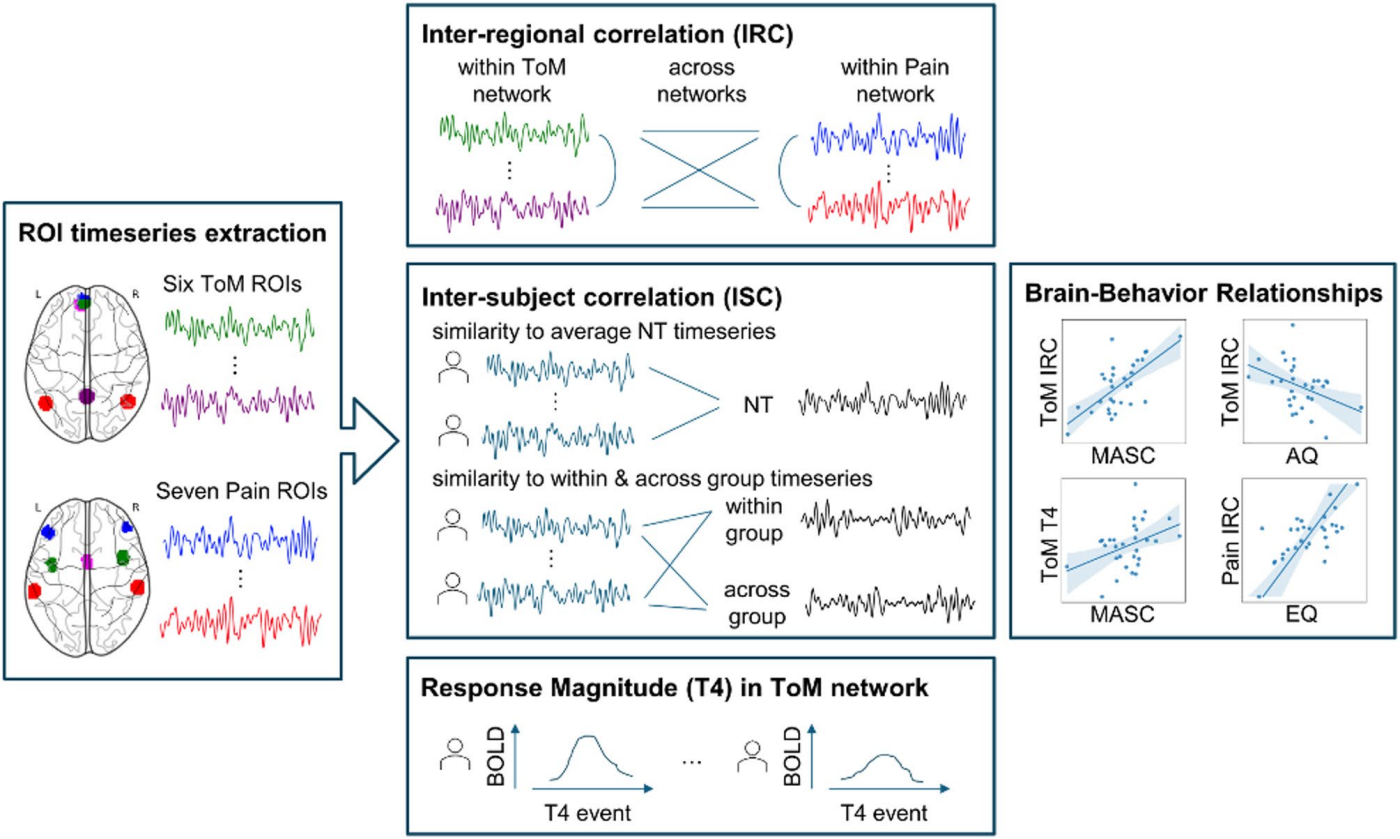
Workflow for fMRI data analyses. ROI time series were extracted from six ToM and seven Pain regions. Inter-region correlations (IRC) were computed within and across networks. Inter-subject correlations (ISC) assessed similarity to the average NT responses and to within/across-group participants. Response magnitude at the final ToM event (T04 from Richardson et al., [54]) and brain-behavior associations with MASC, AQ, and EQ scores were examined. Abbreviations: AQ, autism spectrum quotient; EQ; empathy quotient; IRC, inter-region correlation, ISC, inter-subject correlation; MASC, movie for the assessment of social cognition; NT, neurotypical; ToM, Theory of Mind; T04, final ToM event

**Fig. 2:**
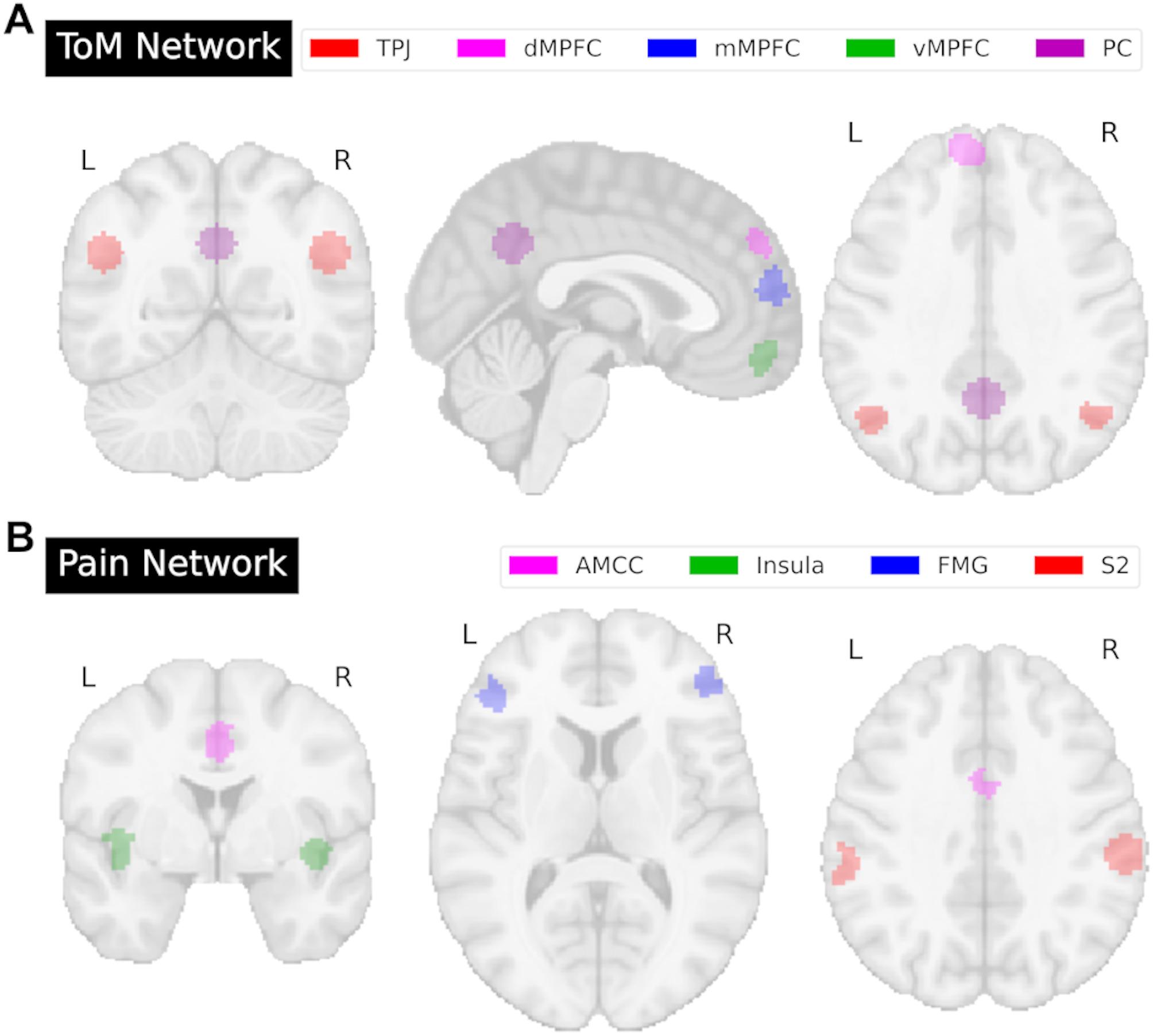
Regions of interest (ROIs) in the Theory of Mind (ToM) and Pain networks. (**A**) Six ToM network ROIs included bilateral temporoparietal junctions (TPJ; red), dorsal medial prefrontal cortex (dMPFC; pink), middle MPFC (mMPFC; blue), ventral MPFC (vMPFC; green), and precuneus (PC; purple). (**B**) Seven Pain network ROIs included anterior mid-cingulate cortex (AMCC; pink), bilateral insula (green), bilateral middle frontal gyrus (MFG; blue), and bilateral secondary sensory cortex (S2; red). Axial, coronal, and sagittal views are shown in MNI space

The current study aimed to provide novel insight into the brain networks underlying inferences about both mental and bodily states in autistic as compared to neurotypical participants using an established passive movie-watching paradigm. We hypothesized that autistic participants would show altered functional specialization of the two brain networks, specifically in the ToM network, with relatively typical (i.e., similar) responses in the Pain network. For both networks, we characterized the functional specialization (Aim 1), assessed heterogeneity (Aim 2), related similarity to average functional network responses in the neurotypical group to inter-region network correlations (Aim 3), and determined the relationship between functional specialization and social behavior (Aim 4) (see Fig. 1 for an analysis overview, Fig. 2 for ToM and Pain brain network regions). To reduce analytic degrees of freedom and promote reproducibility and transparency, hypotheses and analyses were preregistered on the Open Science Framework (https://osf.io/v3bpc/?view_only=55f692128cd645d8aefc5a7069dfb533).

## Methods

### Participants

#### Demographics

Data were previously collected from autistic individuals with a prior ASD diagnosis and neurotypical comparison groups without ASD diagnosis, across two sites (California Institute of Technology (Caltech) and Indiana University (IU)) between 2017 and 2020. The neurotypical participants were community volunteers matched to the autistic individuals in age, sex, and handedness as closely as possible. The full sample included in this study (prior to any exclusion due to e.g., head motion in the scanner) was composed of 38 autistic and 78 neurotypical participants (i.e., typically developed) (see Table S9 in SI for demographic information on the initial sample). Originally the study aimed to include 100 participants per group, however recruitment ended early due to the COVID-19 pandemic restrictions on research in 2020. The data presented here were part of a larger project including other behavioral and neuroimaging assessments, not relevant to the present study. In short, all participants gave informed consent in line with the Institutional Review Boards at Caltech and IU and were reimbursed for participation. Autistic participants had a prior diagnosis of ASD, Asperger’s Syndrome, or Pervasive Developmental Disorder-Not Otherwise Specified (PDD-NOS). The ADOS-2 Module 4 (Autism Diagnostic Observation Schedule (ADOS) [84]) was administered by research-reliable ADOS administrators to confirm diagnoses at both sites, and autistic participants enrolled at Caltech additionally had their diagnosis confirmed by expert clinical diagnosis. Participants were included if scores were within 1 point of the total diagnostic cutoff using the Hus and Lord [85] revised scoring algorithm (i.e., Social Affect + Restricted Repetitive Behaviors > = 7) Scores ranged from 8 to 27 (*m* = 13.54, *sd* = 4.34). Exclusion criteria included for example, a full-scale IQ (FSIQ) below 80, a history of moderate to severe head injury, epilepsy, schizophrenia, bipolar disorder, and the use of antipsychotic medications or hallucinogenic drugs. Autistic traits were also evaluated across both groups with the Autism Spectrum Quotient (AQ) [86]. The AQ is a 50-item self-report questionnaire assessing five domains associated with ASD (social skills, communication skills, imagination, attention to detail, and attention switching/tolerance of change). Scores range from 0 to 50 and are continuously distributed in the general population. A cutoff of 26 demonstrates a sensitivity of 0.95, specificity of 0.52, positive predictive value of 0.84 and negative predictive value of 0.78 [87]. Intellectual functioning was assessed using the Wechsler Abbreviated Scale of Intelligence [88].

#### Socio-cognitive characterization

An overall measure of empathy, encompassing cognitive, social, and emotional domains, was assessed using the Empathy Quotient questionnaire (EQ) [89], a 60-item self-report measure with scores ranging from 0 to 120.

The ability to reason about others’ mental states (ToM) was assessed with the Movie for the Assessment of Social Cognition (MASC) [90]. In this task, participants watch a short 15-minute film about four characters getting together for a dinner party and answer questions referring to the actors’ mental states — feelings, thoughts, and intentions, at multiple times during the movie. Importantly, the MASC does not have ceiling effects in neurotypical populations [90], is sensitive to social difficulties in autistic adults [90–94] and predicts behavioral intervention outcomes on brain function in ASD [95].

### fMRI data

#### Acquisition

We acquired MRI images using identical Siemens 3T Magnetom Prisma Fit scanners (Siemens Medical Solutions, Natick, MA) with 64-channel head receive arrays using identical acquisition sequences at the two sites (Caltech and IU). Scanner software versions used were VE11B (IU) and VE11C (CAL, and the last five participants at IU). High-resolution images of the whole brain were acquired as anatomical references using a multi-echo MPRAGE Sequence (0.9 mm isotropic voxel size; TR = 2.55 s; TEs = 1.63 ms, 3.45 ms, 5.27 ms, 7.09 ms; TI = 1,150 ms). During functional scans, T2*-weighted multiband echo planar imaging (EPI) data were acquired (TR = 720ms; TE = 30 ms; flip angle = 50; 2.5 mm isotropic voxels; 60 interleaved slices with whole brain coverage; multi-band acceleration factor = 6 (Multiband EPI sequence version R16, CMRR, University of Minnesota)). Prior to the first functional scan, spin-echo EPI images were acquired in opposite phase-encoding directions (three images each with P-A and A-P phase encoding) with identical geometry to the EPI data (TR = 4.39 s; TE = 37.2 ms; flip angle = 90) to be used as a fieldmap to correct EPI distortions.

#### Movie stimulus

While in the scanner, participants passively watched a short movie previously used in fMRI research on the neural basis of social cognition [96]. “Partly Cloudy” is a short, animated movie that includes events evoking the mental states and physical sensations of the characters. This movie has been validated as activating ToM brain regions and the Pain Matrix in response to mental events and pain events, respectively, across multiple studies and samples [54, 97]. In contrast to previous studies, the movie’s audio was presented to participants via MRI-safe headphones (Sensimetrics S14) and the first 30 sec of the movie (after opening credits) were not included. Note that events relevant for mental or bodily state inferences (identified by previous studies through reverse correlation analyses [54, 97] start after that time, and were thus not affected by the movie difference.

#### MRI preprocessing

Raw DICOM images were converted to NIfTI format and organized according to the BIDS specification ( [98] http://bids.neuroimaging.io/; Tyszka, J. M., (2023) BIDSKIT: BIDS conversion tool, https://pypi.org/project/bidskit/). After conversion, minimal preprocessing was performed using fMRIPrep version 20.0.7 [99] using components from ANTs [100], FSL (v. 5.0.9; FMRIB’s Software Library, www.fmrib.ox.ac.uk/fsl) and Freesurfer (v.6.0.1) [101]. Briefly, anatomical images were bias-corrected, skull-stripped, segmented, and normalized via nonlinear registration to MNI space (2 mm MNI152NLin6Asym). Functional scans underwent rigid-body motion correction, fieldmap-based distortion correction, and coregistration to the anatomical reference scan, and confound regressors (head motion parameters, CSF, WM, and whole-brain global signal etc.) were computed. Due to the short TR (0.72 s), functional data were not slice-time corrected. The complete details on preprocessing are included in SI, with a boilerplate text generated by fMRIPrep.

#### Denoising and spatial smoothing

After minimal preprocessing, we applied spatial smoothing (5 mm FWHM Gaussian kernel), temporal regression with five principal components from aCompCor regression provided by FMRIPrep, motion artifact timepoint interpolation (see below for details on artifact timepoint definition) and high-pass filtering (100 s cut-off, Butterworth filter as implemented in NiLearn (RRID: SCR_001362) to each voxel, following procedures in Richardson et al., [54] closely. Timecourses were z-scored.

#### MRI data quality exclusion criteria

To assess data quality across data collected in the overall project’s sample (not just data presented here), MRI images were processed with MRIQC (v0.15.2) [102] for initial quality assessment using the functional image quality metrics (IQMs) FWHM avg, SNR, TSNR, DVARS std, and GSR. Interquartile range outliers on these IQMs (the median for the data site plus or minus 1.5 times the IQR for the IQM for the data site) were flagged for manual review by two researchers (L.B., D.K.). Following review, flagged scans were excluded from further analyses if necessary. Further, all minimal preprocessing outputs (fMRIPrep, see above) were visually inspected for data and registration quality following the strategy outlined in Byrge et al., [77].

#### Assessment of in-scanner head motion

In-scanner head motion was quantified using filtered framewise displacement (FDfilt4). Following Power et al., [103] and Byrge & Kennedy [104], framewise displacement at each timepoint was computed as the sum of the (absolute) backward differences over four timepoints of the head movement traces calculated by fMRIPrep, which were filtered using a Butterworth band filter to exclude respiratory frequencies.

#### Motion artifact timepoint detection

Timepoints with FDfilt4 exceeding 0.5 mm were considered motion artifact timepoints; the two timepoints before and after motion artifact timepoints were also treated as motion artifact timepoints. Motion artifact timepoints were censored (i.e., treated as missing data) in the outlined analyses on a subject-wise level.

#### Subject-wise exclusion due to head motion

Similar to the strategy developed and outlined in Byrge et al., [77], we excluded all scans with overall excessive motion identified by mean FDfilt4 exceeding the median plus 1.5 times the IQR of the mean FDfilt4 across all scans in the overall project (including scans from participants not included in the current analyses), computed separately at each site (mean FDfilt4 > 0.4808 for IU, mean FDfilt4 > 0.5625 for Caltech). In addition, we excluded participants if the number of motion artifact timepoints (FDfilt4 > 0.5 mm) exceeded one third of the scan, following Richardson et al., [54].

#### Comparing head motion across groups

We compared mean FDfilt4 (mFDfilt4) and the number of motion artifact timepoints (nMotionTR) across groups with independent samples t-tests. Because the two groups differed in these metrics, we removed subjects with the highest and lowest values in each group until levels of head motion were similar across the groups (following the preregistered analysis plan). The goal was to maintain the largest number of subjects possible in each group while reducing group differences in head motion. To nevertheless account for the effects of motion, we used mFDfilt4 (computed over all TR) as an approximation of head motion in statistical analyses (‘head motion’ covariate).

#### Exploratory analyses on further matched groups

In addition to the analyses including the sample outlined above, we performed exploratory analyses using more conservative matches of potentially confounding variables, with the goal of having the same sample size in each group (retaining the maximum number of ASD participants, *n* = 34) without mean differences in head motion, IQ, age, sex (*p* > 0.05) (see Table 1).

**Table 1:** Participant characteristics - full matched sample (after motion exclusion) and closely matched sample

	FULL SAMPLE (*N* = 107)					MATCHED SAMPLE (*N* = 68)				
	ASD (*N* = 34)	NT (*N* = 73)	chi^2^ / t	*p*	*d*	ASD (*N* = 34)	NT (*N* = 34)	chi^2^ / t	*p*	*d*
*Sex* *(F/M)*	11/23	23/50	0.01	0.93	0.01	11/23	11/23	0	1	0
*Hand* *(R/L/A)*	27/7/0	64/7/2	3.26	0.20	0.18	27/7/0	31/3/0	1.88	0.17	0.17
*Age* *(years)*	21–46 27.1 (5.4)	19–55 27.4 (7.2)	1.03	0.31	0.04	21–46 27.1 (5.4)	20–44 28.8 (6.1)	1.08	0.28	0.3
*FSIQ*	*n* = 33	*n* = 68				*n* = 33	*n* = 34			
	84–142 112.9 (13.2)	87–136 109.9 (10.7)	0.87	0.39	0.26	84–142 112.9 (13.2)	92–136 109.3 (11.9)	0.64	0.53	0.29
*AQ*	*n* = 33	*n* = 68				*n* = 33	*n* = 34			
	10–48 27.9 (9.2)	4–40 16.4 (6.8)	7.04	<2e-16	1.51	10–48 27.9 (9.2)	4–23 13.3 (4.9)	7.61	1.6e-10	1.98
*MASC*	*n* = 27	*n* = 64				*n* = 27	*n* = 34			
	7–42 29.4 (8.8)	27–43 35.5 (3.5)	5.41	5e-07	1.11	7–42 29.4 (8.8)	27–41 35.4 (3.6)	4.11	0.0001	0.94
*EQ*	*n* = 32	*n* = 68				*n* = 32	*n* = 34			
	9–90 33.1 (17.1)	6–75 47.3 (14.1)	5.14	1.3e-06	0.94	9–90 33.1 (17.1)	30–75 52.2 (11.4)	5.45	9.1e-7	1.32
*exclTR*	0–133 52.6 (37.7)	0–146 37.1 (41.3)	1.86	0.07	0.39	0–133 52.6 (37.7)	0–146 47.3 (43.1)	0.54	0.59	0.13
*mFD*	0.1–0.4 0.2 (0.08)	0.1–0.4 0.2 (0.08)	1.49	0.14	0.31	0.1–0.4 0.2 (0.08)	0.1–0.4 0.2 (0.08)	0.03	0.98	0.01

Note: Values represent range, mean and standard deviation (SD); all t-tests reflect group differences after controlling for group*site interactions

*Abbreviations*: ASD, autism spectrum disorder; AQ, Autism Spectrum Quotient; d, Cohen’s d; EQ, Empathy Quotient; exclTR, number of excluded TR; F, female; FSIQ, Full Scale Intelligence Quotient; Hand, Handedness; M, male; MASC, Movie for the Assessment of Social Cognition; mFD, mean framewise displacement across all TR; n/N, number of subjects; NT, neurotypical; TR, repetition time

#### Regions of interest

We used regions of interest (ROIs, see Fig. 2, Fig. 1) of the ToM and Pain network comprising 9 mm spheres around peak voxels from prior group analyses based on responses to the “Partly Cloudy” movie in an independent sample of neurotypical adult participants (*n* = 20), described in Richardson et al., [54], including six ToM ROIs (dorsal (dMPFC), ventral (vMPFC) and middle MPFC (mMPFC), precuneus (PC), right and left temporoparietal junction regions (rTPJ, lTPJ)) and seven Pain ROIs (anterior mid-cingulate cortex (AMCC), right and left insula (rIns, lIns), middle frontal gyrus (rMFG, lMFG) and secondary sensory cortex (rS2, lS2)).

#### Data analyses

*Inter-regional correlation analyses (IRC).* Inter-regional correlation analyses were conducted to assess functional connectivity (FC) within and between the ToM and Pain networks (see Fig. 1 for analyses workflow). The timecourse for a given ROI was obtained by averaging the timecourses across all voxels in the ROI. Motion artifact timepoints were subsequently excluded per subject. Averaged ROI time series were then correlated across ROIs *within* and *across* networks using Fisher-transformed Pearson’s correlation per subject. To compute *within-ToM* correlations, we used the average correlation from each ToM ROI to every other ToM ROI. To compute *within-Pain* correlations, we likewise used the average correlation from each Pain ROI to every other Pain ROI. To compute *across-ToM/Pain* network correlations, we calculated the average correlation from each ToM ROI to each Pain ROI (following procedures in [54, 105]). IRC analyses were based on the complete preprocessed stimulus timeseries duration (as opposed to certain events) following closely procedures outlined in the relevant prior work using the same stimulus and analysis approach [54], and maximizing the amount of data used for analyses.

#### Similarity of timecourse response to average response in neurotypical group

We computed the similarity of each subject’s network timecourse to the average neurotypical network timecourse response (similarityNT) using Fisher-transformed Pearson’s correlation. To compute similarity in each network (*similarityNT-ToM* and *similarityNT-Pain*), we first calculated the average response timecourse across all ROIs of the network per subject and then correlated a subject’s network timecourse with the NT group average network timecourse. For NT participants, we used a leave-one-out (LOO) approach when comparing to the group average (i.e., each NT participant’s timecourse was left out of the group average timecourse, for estimating the similarity of their response to the group average response).

#### Similarity of timecourse response to within-group versus across-group participants

First, we computed the similarity of each subject’s network timecourse to every other participant’s network timecourse response, *within-group* and *across-group*. Second, we averaged all within-group and across-group correlations per subject to create two scores per subject, separately for each network [*within-groupToM*,* across-groupToM*,* within-groupPain*,* across-groupPain*].

#### Response magnitude to last ToM event (“T04”) in ToM ROIs

Prior research found that, among children, response magnitude to the last ToM event in ‘Partly Cloudy’ correlated with performance on a ToM behavioral battery, controlling for age [54]. In this scene (“T04”), the stork character “Pecks” puts on gear to explain why he left in a previous scene. To estimate the response magnitude to T04, we averaged timecourses to create a single ToM network timecourse and extracted the response magnitude value from the peak timepoint from this event, for each participant, as described in Richardson et al., [54]. Due to TR differences (0.72s vs. 2s), the peak timepoint included multiple TRs.

### Statistical analyses

All analyses included null hypothesis testing and used a significance threshold of *p* < 0.05. Linear regression analyses were conducted to examine the effect of Group (ASD vs. NT) on inter-region correlations (IRC) within-network and across-network, as well as association between IRC with the average neurotypical timeseries similarity and socio-cognitive variables, while controlling for motion (mFD), age, sex, FSIQ, and site. Paired t-tests were conducted to test whether timecourse responses in each network were more similar within-group (ASD-ASD, NT-NT) as compared to across groups (ASD-NT, NT-ASD). Welch two-sample t-tests were conducted to test whether timecourse responses in each network were more similar across NT participants as compared to ASD participants (i.e., whether response timecourses are more heterogeneous across ASD individuals). We added effect size estimations using Cohen’s d, as additional quantification not outlined in the preregistration. Given the use of individually modeled comparisons to test distinct hypotheses (as opposed to multiple tests of a single family of hypotheses), we did not perform post-hoc multiple comparison correction across the tested hypotheses, except when we applied Bonferroni correction for the number of tests when correlating a neural measure (within-ToM IRC) with two behavioral tests (MASC, AQ). For the hypotheses predicting the absence of a group effect (e.g., for the Pain network), we estimated the evidence for the null hypothesis (over Alternative hypothesis) if *p* > 0.05 with Bayes factor by calculating the respective Bayesian analyses as implemented in JASP with default parameters. See code for further detail of all analyses.

## Results

### Participants

#### Exclusions

Following preregistered exclusion criteria, we excluded seven participants (ASD *n* = 3, 2 from Caltech site; NT *n* = 4, 3 from Caltech site)) from the initial sample (*n* = 116) due to excessive head motion (i.e., more than one third of the complete timeseries identified as motion artifact timepoints). For the remaining sample, groups significantly differed in the total number of excluded timepoints, thus, we excluded two more participants (one per group, with highest/lowest motion) to arrive at a sample not significantly different in motion - as outlined in the preregistration. The remaining “full sample” (*n* = 107) included 34 autistic and 73 neurotypical (NT) participants (see Table 1).

#### Demographics

Groups did not differ in handedness, sex, intellectual functioning, or age (see Table 1 for detailed statistics), however, there was a significant main effect of site [CAL, IU] for both age and FSIQ, and a site-by-group interaction on age. We thus included main effects of group, age, sex, FSIQ and an interaction term for group*site in the initial regressions and removed non-significant interaction terms for final models (see SI Tables S1-4 for models including interaction terms). Of note, these analyses and respective decisions were performed prior to the fMRI hypothesis testing, as outlined in the preregistration.

#### Socio-cognitive functioning

**Fig. 3:**
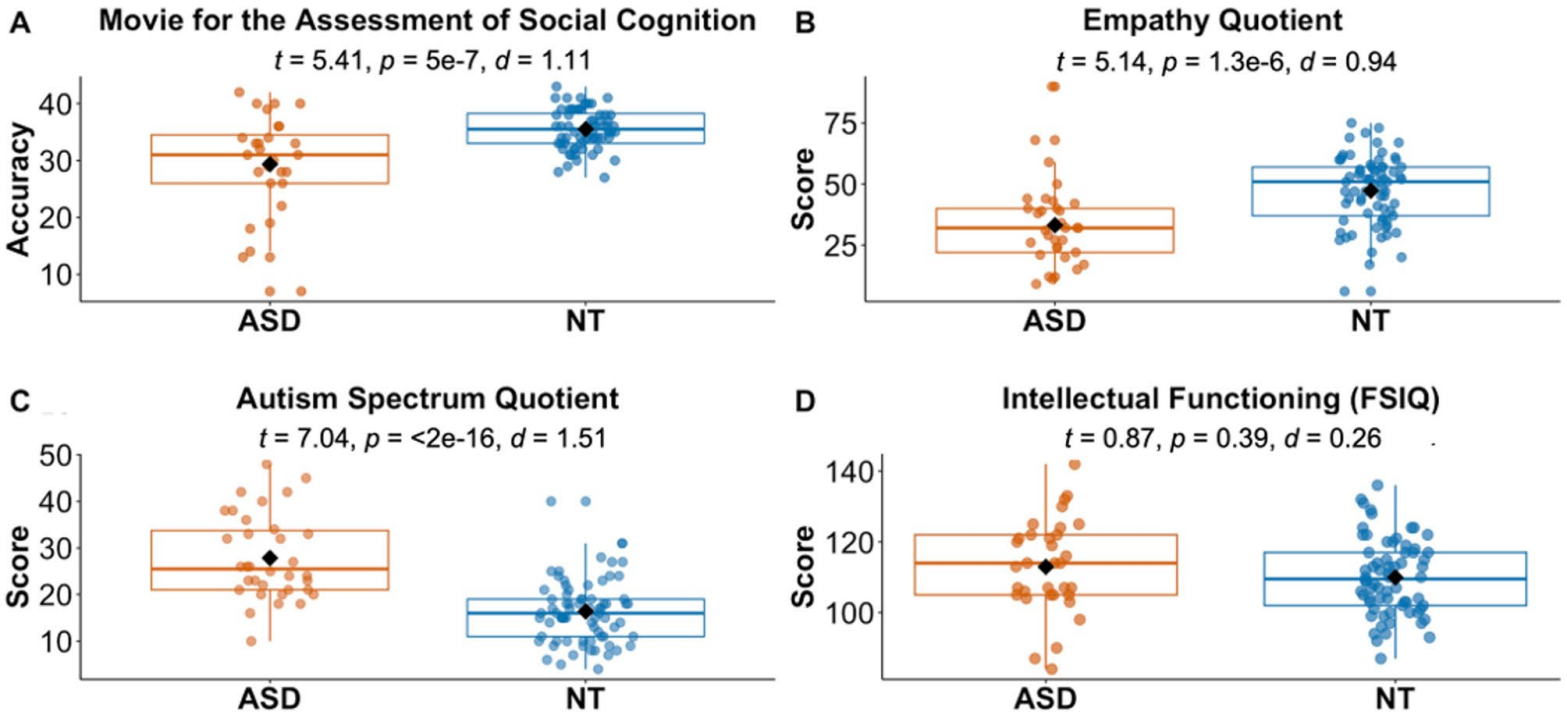
Demographic and socio-cognitive characteristics by group. Boxplots showing (**A**) ToM Performance: total accuracy score for the Movie for the Assessment of Social Cognition (MASC), (**B**) self-reported Empathy: total score for the Empathy Quotient (EQ), (**C**) self-reported ASD symptoms: total score for the Autism Spectrum Quotient (AQ), and (**D**) Intellectual functioning: Full Scale Intelligence Quotient (FSIQ) across groups (ASD: orange, NT: blue). Diamond markers indicate mean values while horizontal lines within each box represent median values. *Abbreviations*: ASD, autism spectrum disorder; d, cohen’s d (effect size); NT, neurotypical; p, *p* value; ToM, Theory of Mind; t, t statistic

As expected, the autistic group showed significantly higher AQ scores (β = 0.59, *SE* = 0.84, *t* = 7.04, *p* = 2.19e-10, *d* = 1.51) and significantly lower scores on the MASC (β = -0.50, *SE* = 0.63, *t* = -5.41, *p* = 5e-07, *d* = 1.11) and EQ (β = -0.47, *SE* = 1.62, *t* = -5.14, *p* = 1.32e-06, *d* = 0.94), indicating higher levels of autistic symptoms, reduced mental state inference performance and lower levels of general empathy, respectively. There was an additional significant main effect of site for the AQ (β = 0.15, *SE* = 0.84, *t* = 1.73, *p* = 0.09, *d* = 0.35), with higher AQ values overall at the IU site. There were further significant interactions of site and group for the MASC (β = 0.23, *SE* = 0.63, *t* = 2.29, *p* = 0.02, *d* = 0.33) and the EQ (β = 0.23, *SE* = 1.62, *t* = 2.29, *p* = 0.02, *d* = 0.35) (see Fig. 3). For both measures, the group difference was more pronounced at the Caltech site.

### Exploratory analyses with more closely matched samples

In addition to the main analyses on the full sample, we also performed exploratory analyses, repeating all preregistered analyses in a very closely matched sample with equal group size (*n* = 34 per group, total *n* = 68), for sex, age, FSIQ and with an emphasis on minimizing and matching head motion (see Table 1). While the full sample was not statistically different in respective variables between groups based on (see Table 1) the criteria defined in the preregistration (*p* value), effect sizes especially for the motion variables were of moderate size (excludedTR: *d* = 0.39; mFD: *d* = 0.310). We hence added exploratory analyses on the more closely matched samples, to ask if the results generalized, and were thus less likely to be affected by potential confounding variables, especially motion. The pattern of results (including the group effects) on the full and matched samples were largely consistent (see SI Tables S5-8). Notable differences are highlighted in specific results sections. We discuss implications in detail in the discussion section.

### IRC within and across networks in ASD

**Fig. 4:**
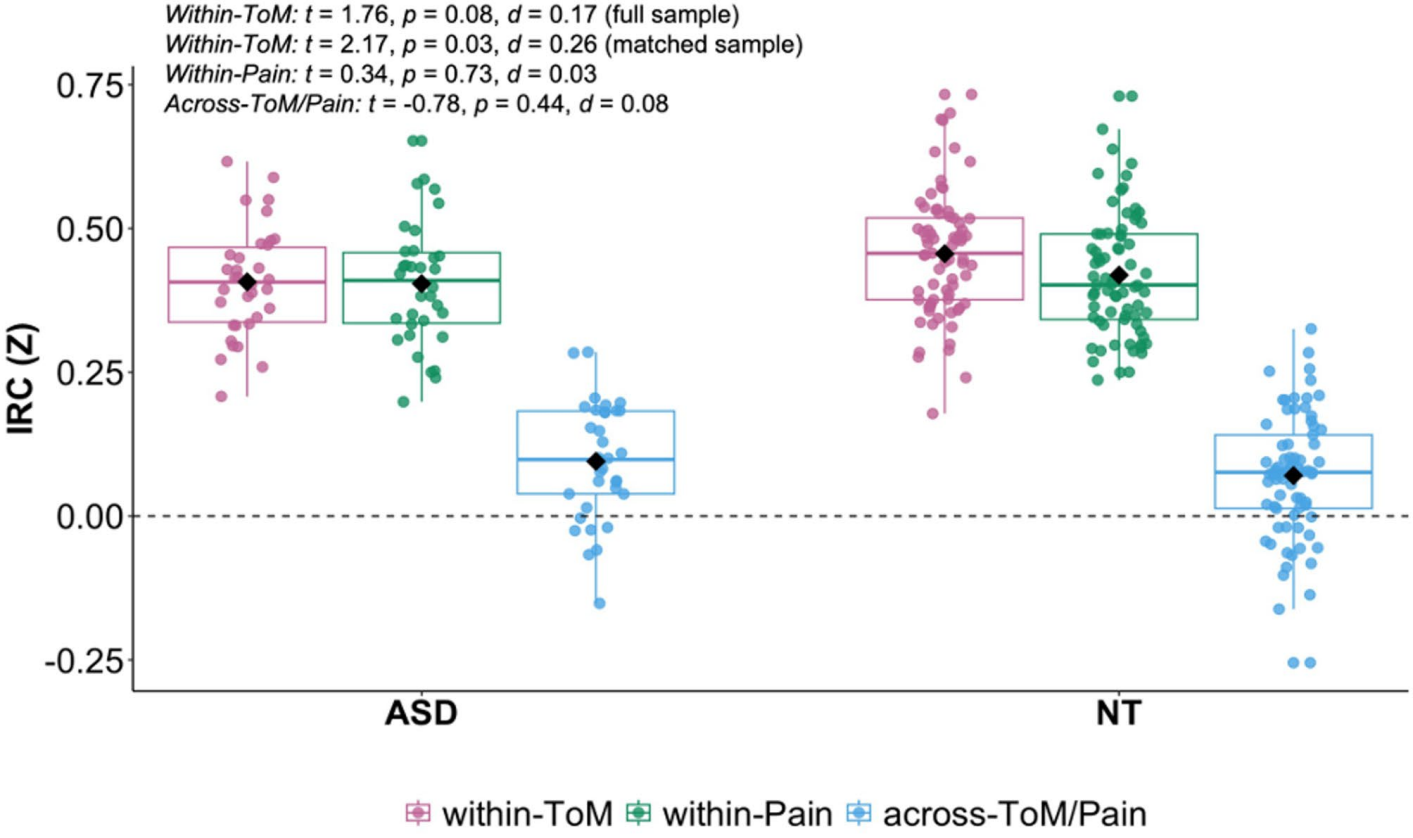
Functional Specialization of social brain networks. Inter-region correlation boxplots *within-* (ToM: pink, Pain: green) and *across-* (blue) networks as a function of group (left: ASD, right: NT). Diamond markers indicate mean values while horizontal lines within each box represent median values. *Abbreviations*: ASD, autism spectrum disorder; d, cohen’s d (effect size); IRC, inter-region correlation, NT, neurotypical; p, *p* value; ToM, Theory of Mind; t, t statistic; z, Fisher z-transformed

To characterize the functional specialization of the ToM and Pain networks in autistic as compared to neurotypical participants, we investigated within- and across-network connectivity across groups with inter-regional correlation (IRC) analyses. We hypothesized that the autistic participants would show reduced IRC in the ToM network, as well as less anti-correlated (i.e., negatively correlated) IRC across the ToM and Pain networks, yet similar IRC in the Pain network, as compared to NT participants. In line with our hypothesis, we observed higher correlations between regions within the ToM network in the neurotypical sample (NT: *m* = 0.46, *sd* = 0.11; ASD: *m* = 0.41, *sd* = 0.10) (see Fig. 4). Notably, the effect was overall small: in the full sample the group effect remained numerical (β = 0.18, SE = 0.02, *t* = 1.76, *p* = 0.08, *d* = 0.17), while passing significance threshold for the closely matched sample (β = 0.26, SE = 0.02, *t* = 2.17, *p* = 0.03, *d* = 0.26) (see Table S1 in SI for full model details). Also, in line with our hypothesis, within-Pain IRC was similar across groups (NT: *m* = 0.42, *sd* = 0.11; ASD: *m* = 0.40, *sd* = 0.11; β = 0.03, SE = 0.02, *t* = 0.34, *p* = 0.73, *d* = 0.03). A Bayesian regression analysis suggested that the observed data are 2.16 times more likely under a null model that only contains the covariates than a model containing group as an additional predictor (see Table S10). Correlations of timeseries for regions across the ToM and Pain networks were similar between groups (NT: *m* = 0.07, *sd* = 0.11; ASD: *m* = 0.10, *sd* = 0.10; β = -0.07, SE = 0.02, *t* = -0.78, *p* = 0.44, *d* = 0.08), contrary to our hypothesis. Analyses further revealed a significant main effect of age across both groups (β = -0.24, SE = 0.002, *t* = -2.01, *p* = 0.05, *d* = -0.19), with older individuals exhibiting generally lower connectivity in the Pain network.

### Reduced similarity to the average neurotypical network response in ASD

We assessed similarity to the average neurotypical timecourse response across groups and hypothesized reduced similarity in the ToM network, but no difference in the Pain network, for autistic participants.

**Fig. 5:**
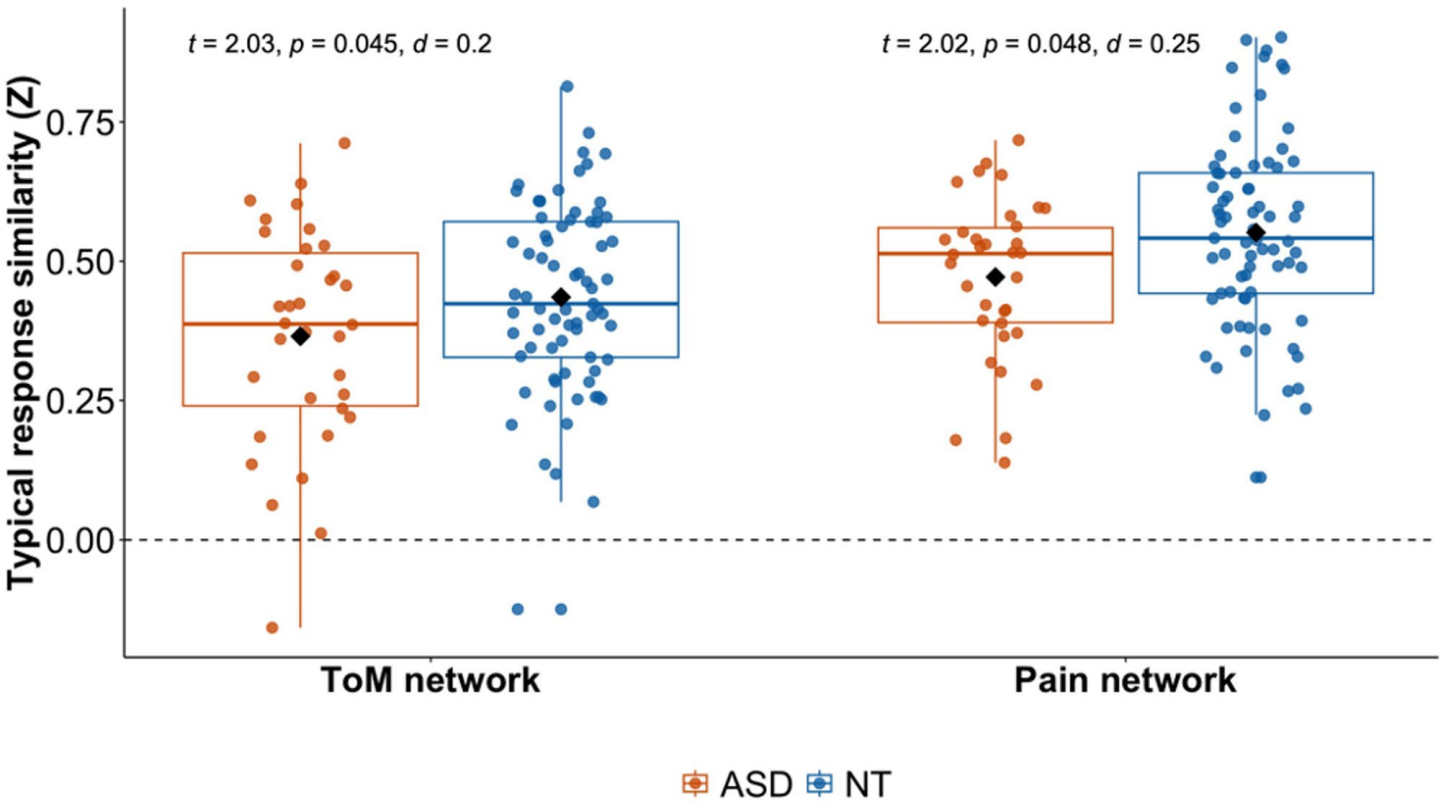
Similarity to the average typical (NT) timecourse for each network per group. Typical response similarity of the within-ToM (left) and within-Pain (right) network values per group (ASD: orange, NT: blue). Diamond markers indicate mean values while horizontal lines within each box represent median values. *Abbreviations*: ASD, autism spectrum disorder; d, cohen’s d (effect size); IRC, inter-region correlation, NT, neurotypical; p, *p* value; ToM, Theory of Mind; t, t statistic; z, Fisher z-transformed

Overall, we found small effects of group in both the full and closely matched sample: for the ToM network, reduced similarity was significant in the full sample ((NT: *m* = 0.44, *sd* = 0.17; ASD: *m* = 0.37, *sd* = 0.20; β = 0.19, SE = 0.04, *t* = 2.03, *p* = 0.045, *d* = 0.2), and showed a trend for the closely matched sample (NT: *m* = 0.43, *sd* = 0.16; ASD: *m* = 0.37, *sd* = 0.20; β = 0.21, SE = 0.04, *t* = 1.8, *p* = 0.08, *d* = 0.22) (see Fig. 5 and SI Table S2 for full model details). For the Pain network, similarity was reduced significantly in the matched sample (NT: *m* = 0.53, *sd* = 0.16; ASD: *m* = 0.47, *sd* = 0.15; β = 0.24, SE = 0.04, *t* = 2.02, *p* = 0.048, *d* = 0.25) and trend level only for the full sample (NT: *m* = 0.55, *sd* = 0.17; ASD: *m* = 0.47, *sd* = 0.15; β = 0.17, SE = 0.03, *t* = 1.82, *p* = 0.07, *d* = 0.18). Note that the effect sizes of the group differences were small in general, regardless of their significance values. There was an additional main effect of motion in both networks (ToM: β = -0.27, SE = 0.2, *t* = -2.88, *p* = 0.005, *d* = 0.28; Pain: β = -0.34, SE = 0.19, *t* = -3.59, *p* = 0.0005, *d* = 0.03) across all participants (and for the matched sample, see SI Table S6). Increased motion was associated with lower timecourse similarity. In addition, age was related to similarity in the ToM network with reduced timecourse similarity in older participants (ToM: β = -0.27, SE = 0.003, *t* = -2.45, *p* = 0.02, *d* = -0.24).

### Heterogeneity of social brain networks

To assess the heterogeneity of the ToM and Pain network responses between groups, we compared within- and across-group similarity per network. We hypothesized that both groups would show greater within-group as compared to across-group similarity of network responses in the ToM network, but similar within- and across-group similarity for the Pain network. We further hypothesized that within-group similarity would be reduced (indicating more heterogeneous response timecourses) in autistic participants only in the ToM network and similar between groups for the Pain network.

#### Different patterns of within- versus across-group similarity between groups

**Fig. 6:**
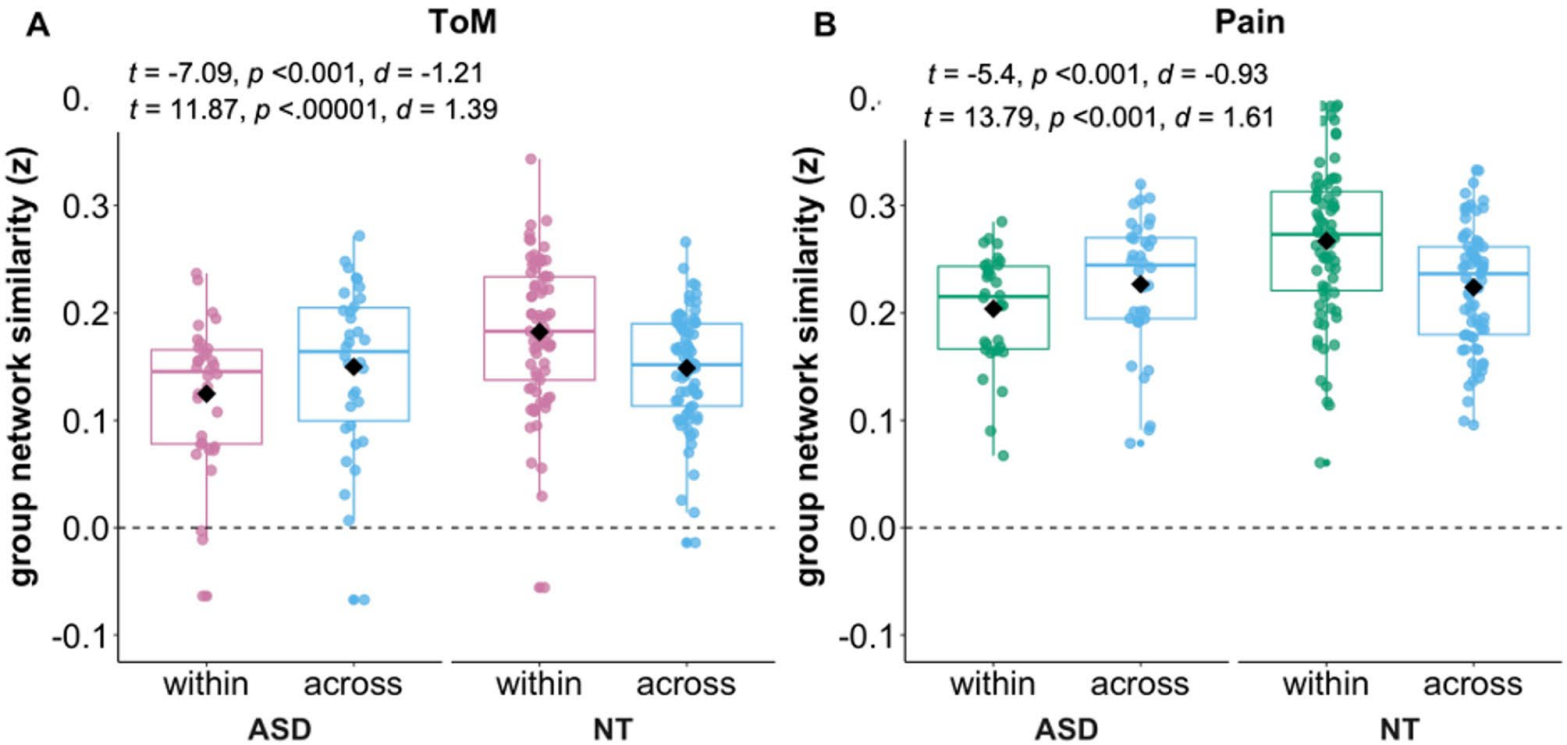
Within-group and across-group network similarity. Boxplots showing within- and across-group ToM (**A**) and Pain (**B**) network similarity values per group. Diamond markers indicate mean values while horizontal lines within each box represent median values. *Abbreviations*: ASD, autism spectrum disorder; d, cohen’s d (effect size); NT, neurotypical; p, *p* value; ToM, Theory of Mind; t, t statistic; z, Fisher z-transformed

Comparing within- versus across-group similarity for each group separately (see Fig. 6), we found that the timecourse responses in both networks were significantly *more* similar within-group as compared to across-group in the NT group (ToM: mean difference: 0.03 (95% CI [0.03, 0.04]), *t*(72) = 11.87, *p* < 0.0001, *d* = 1.39; Pain: mean difference: 0.04 (95% CI [0.04, 0.05]), *t*(72) = 13.79, *p* < 0.001, *d* = 1.61). In contrast, we found significantly *less* similar timecourse responses within-group as compared to across-group in the autistic group (ToM: mean difference: -0.02 (95% CI [-0.03, -0.02]), *t*(33) = -7.07, *p* < 0.001, *d* = -1.21; Pain: mean difference: -0.02 (95% CI [-0.03, -0.01]), *t*(33) = -5.4001, *p* < 0.001, *d* = -0.93). In other words, the autistic group was less similar to the autistic average (and more similar to the neurotypical average), indicating more idiosyncratic responses.

#### Increased heterogeneity across autistic relative to neurotypical participants

Comparing within-group similarity between groups, we found that the timecourse responses were significantly more similar in the NT group as compared to the autistic group for both networks (ToM, NT: *m* = 0.18, *sd* = 0.07, ASD: *m* = 0.12, *sd* = 0.07, mean difference: 0.06 (95% CI: [-0.09, -0.03]), *t*(65.1) = -4.13, *p* = 0.0001, *d* = -0.86; Pain, NT: *m* = 0.27, *sd* = 0.07, ASD: Pain: *m* = 0.2, *sd* = 0.05, mean difference: 0.06 (95% CI: [-0.09, -0.04]), *t*(87.797) = -5.13, *p* = 0.000002, *d* = -1.06), indicating greater heterogeneity of responses in the ASD group.

### The relationship between functional specialization and network response similarity

Next, we investigated the relationship between the similarity to the average NT network responses and inter-regional network correlations, similar to Richardson et al., [54]. We hypothesized that for the autistic participants, the relationship between similarity to the average NT network response and the inter-region correlations within-ToM network and across-ToM/Pain networks would be reduced as compared to the NT participants. We further hypothesized that these correlations would differ as a function of group (NT > ASD). For the Pain network, we further hypothesized that the correlations between similarity to the average NT network response and the inter-region correlation within-Pain and across-ToM/Pain networks would be similar and not differ as a function of group.

**Fig. 7:**
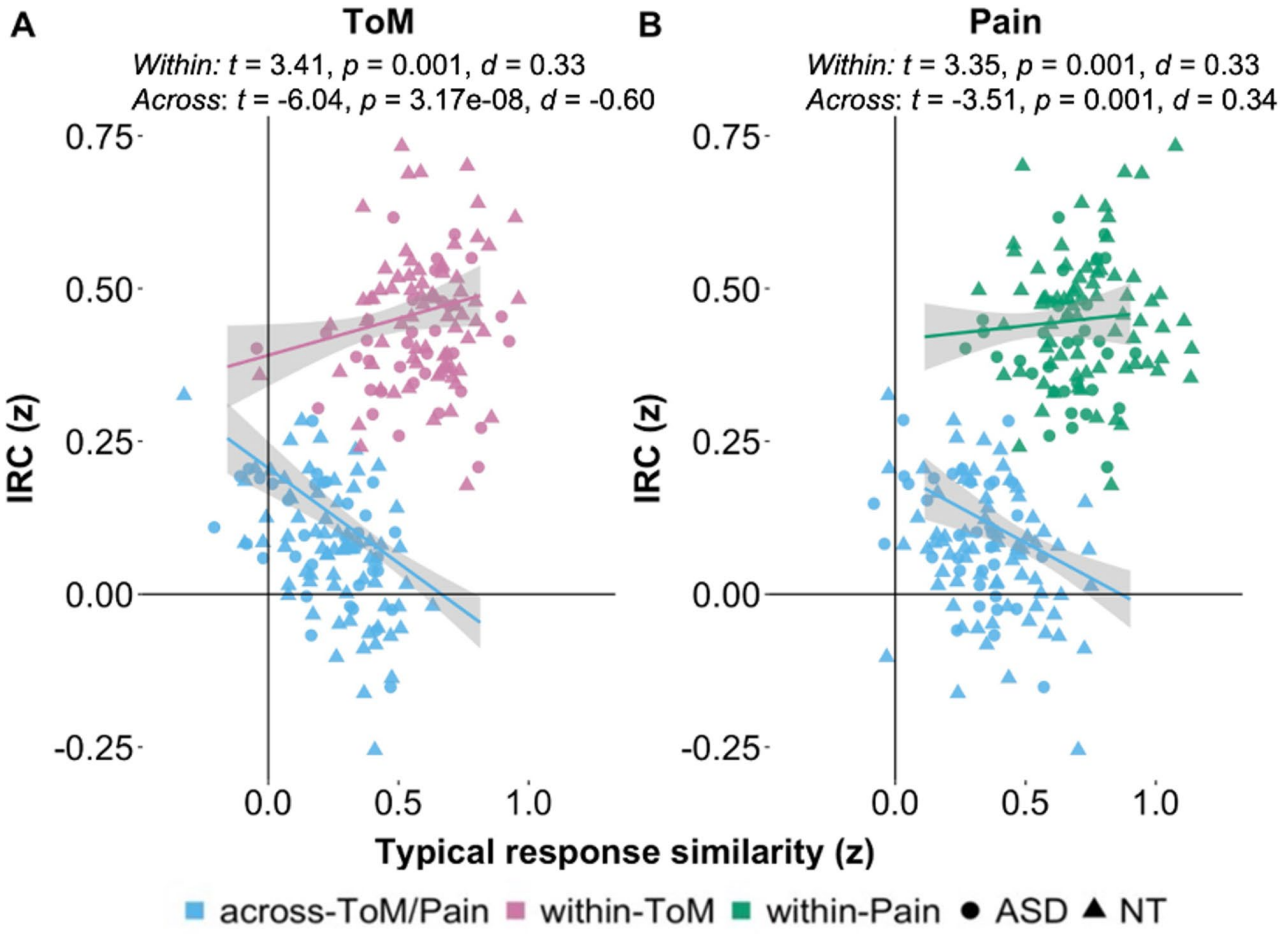
Relating typical response functional similarity to inter-region correlations. In both networks (**A**: ToM; **B**: Pain), typical response similarity (i.e., how correlated each participant’s timecourse was to the average NT timecourse (ISC, x-axis)), was positively correlated with within-network IRC (IRC, y-axis) (pink (ToM network), green (Pain network)) and negatively correlated with across-network IRC (blue). Scatterplots show values for all participants per group (circles = ASD, triangles = NT). Abbreviations: ASD, autism spectrum disorder; d, cohen’s d (effect size); ISC; inter-subject correlation; IRC, inter-region correlation, NT, neurotypical; p, *p* value; ToM, Theory of Mind; t, t statistic; z, Fisher z-transformed

For the ToM network, we found a significant positive association between functional similarity to the average NT timecourse and within-network correlations (β = 0.29, SE = 0.14, *t* = 3.41, *p* = 0.001, *d* = 0.33), as well as a negative association with across-network correlations (β = -0.54, SE = 0.15, *t* = -6.04, *p* = 3.17e-08, *d* = -0.60) and no difference in these correlations across groups - against our hypothesis (see Fig. 7 and SI Table S3 for full model details). In other words, across all participants, greater connectivity within the ToM network and weaker connectivity across networks was associated with increased similarity to the average NT timecourse.

For the Pain network, we found similar patterns of results: similarity to the average NT Pain network timecourse was positively correlated with within-Pain IRC (β = 0.33, SE = 0.16, *t* = 3.35, *p* = 0.001, *d* = 0.33), and negatively correlated with across-network IRC (β = -0.36, SE = 0.16, *t* = -3.51, *p* = 0.001, *d* = 0.34). These relationships did not differ as a function of group, in line with our hypothesis.

### Brain-behavior relationships

Lastly, we investigated the relationship between functional specialization in the social brain networks and social behavior and tested for group interaction effects.

**Fig. 8:**
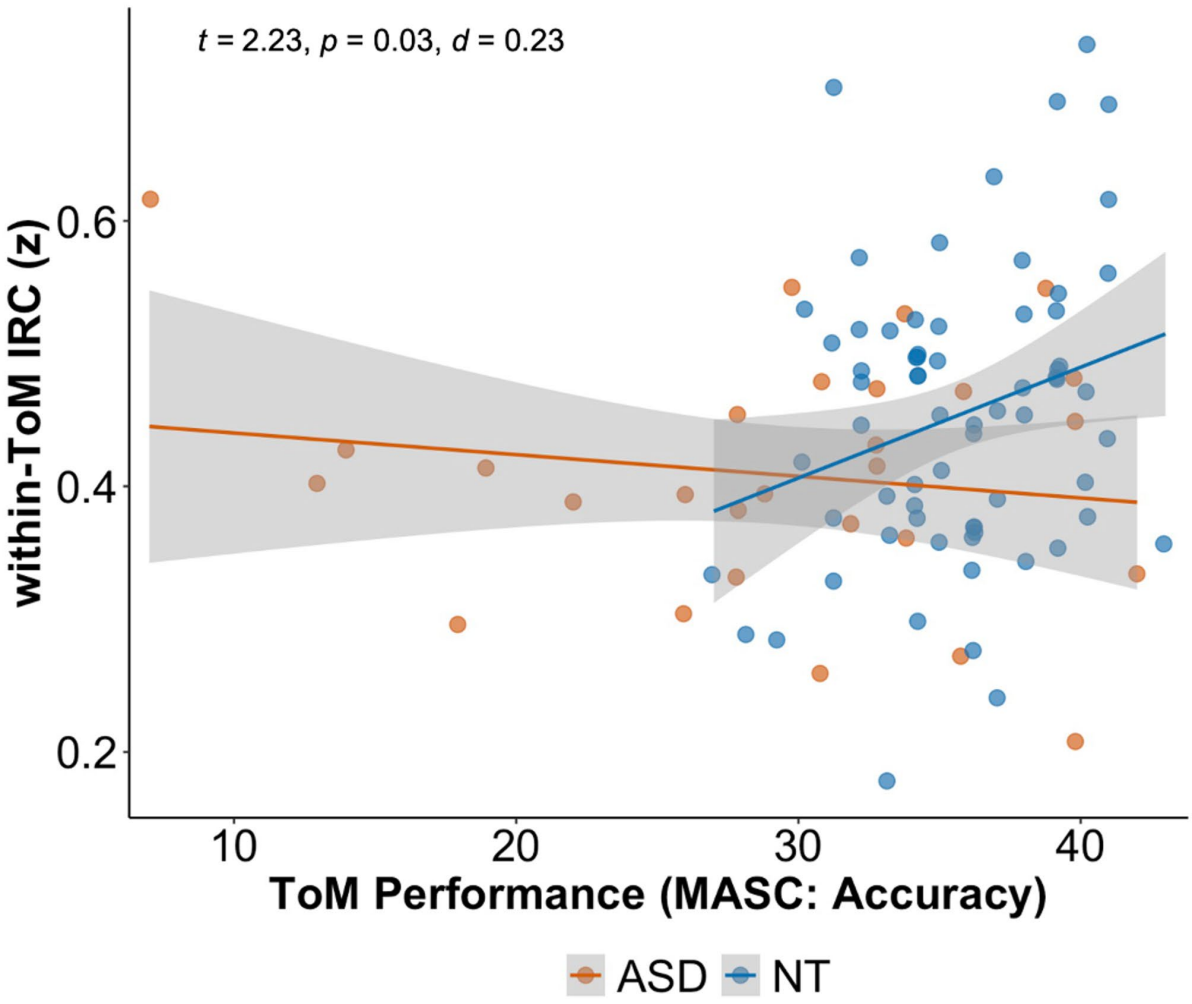
Relationship between within-ToM network inter-region correlations and behavioral performance on the MASC per group. Within-ToM network connectivity (IRC; y-axis), was positively correlated with ToM performance (MASC: Accuracy; x-axis) for NT participants (blue), with no such relationship in ASD participants (orange). *Abbreviations*: ASD, autism spectrum disorder; d, cohen’s d (effect size); IRC, inter-region correlation, NT, neurotypical; MASC, Movie for the Assessment of Social Cognition; p, *p* value; ToM, Theory of Mind; t, t statistic; z, Fisher z-transformed

First, we hypothesized that inter-region correlation in the ToM network would be positively correlated with social inference performance on the MASC task and negatively correlated with levels of autistic symptoms assessed with the AQ. While performance on the MASC was not associated with within-network correlations in the ToM network, there was a significant interaction with the factor group on the brain-behavior relationship (see Fig. 8 and SI Table S4 for full model details and results). Specifically, for NT participants, increased accuracy in mental state inferences correlated with greater within-network connectivity in ToM brain regions (β = 1.63, SE = 0.005, *t* = 2.23, *p* = 0.03, *d* = 0.23), yet not passing conservative multiple comparison correction (alpha = 0.025) for correlating two tests (MASC, AQ) with the same measure. There was no such relationship in ASD (β = -0.14, SE = 0.003, *t* = -0.97, *p* = 0.33, *d* = -0.10). We did not find a significant interaction between group and self-reported levels of autistic symptoms on the AQ (see SI Table S4 for brain-behavior models including non-significant interactions). AQ scores were not related to within-ToM IRC (β = -0.07, SE = 0.001, *t* = -0.71, *p* = 0.48, *d* = -0.07).

Second, we hypothesized that the response magnitude at the peak timepoints of the last ToM event in the movie (as defined by Richardson et al., [54]) would be positively correlated with MASC performance. However, after removing the non-significant interaction between response magnitude and group, we did not find such an association (MASC: β = -0.11, SE = 0.01, *t* = -0.96, *p* = 0.34, *d* = -0.10) nor a group effect.

Third, against our hypothesis, we did not observe a relationship between inter-region correlation in the Pain network and self-reported empathy (EQ) (β = -0.04, SE = 0.001, *t* = -0.36, *p* = 0.72, *d* = -0.04).

## Discussion

The current study aimed to examine the functional specialization, heterogeneity, and brain-behavior relationships of the ToM and Pain networks in autistic compared to neurotypical participants using a passive movie-watching paradigm. Using functional neuroimaging, we investigated whether differences in neural responses underlying thinking about others’ mental states and empathic concerns for others’ bodily feelings might reflect an “imbalance” in social cognition in autism.

**Fig. 9:**
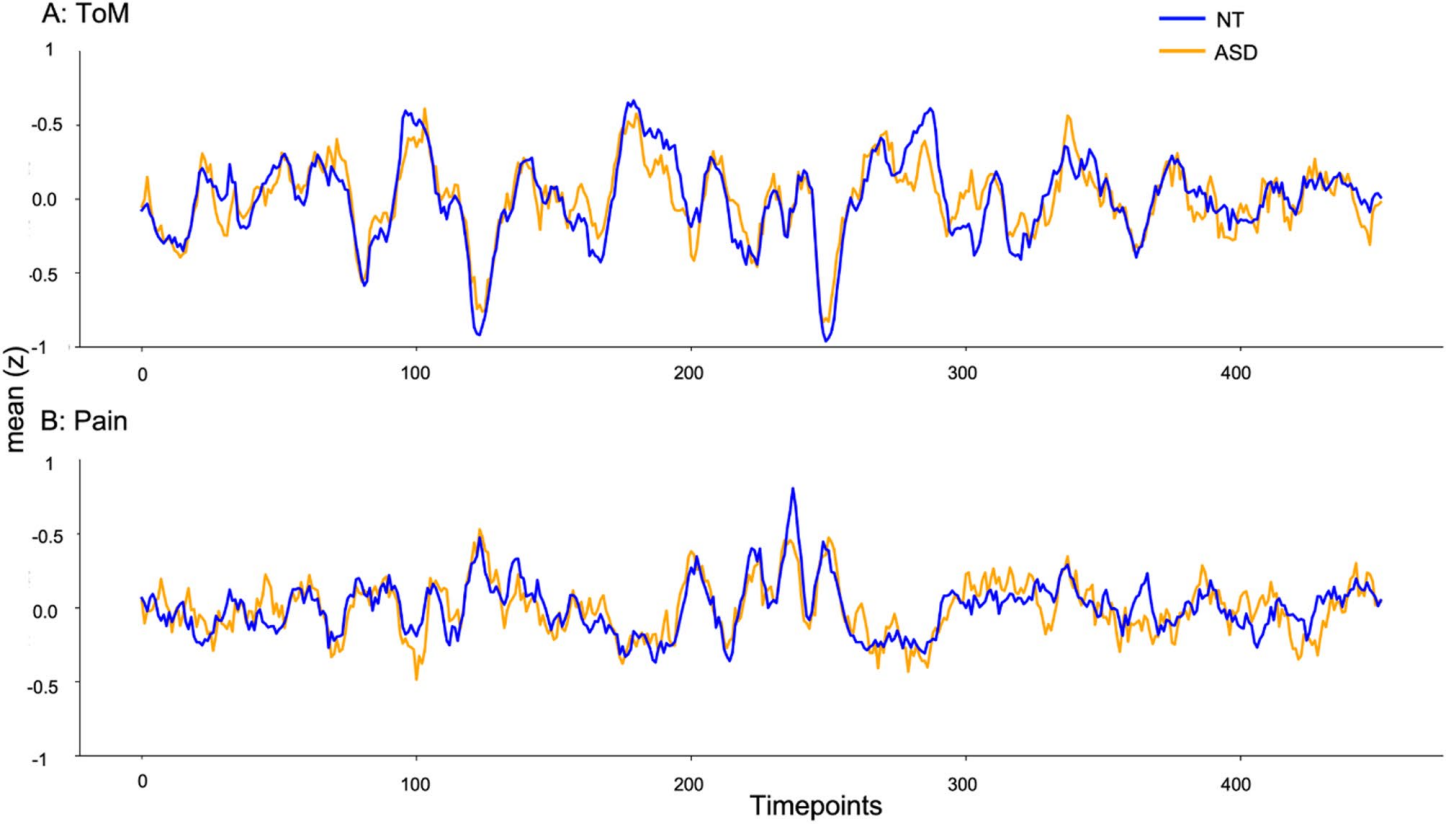
Average timecourse responses in the ToM (**A**, top) and Pain (**B**, bottom) networks across groups (orange: ASD, blue: NT). *Abbreviations*: ASD, autism spectrum disorder; d, cohen’s d (effect size); NT, neurotypical; p, *p* value; ToM, Theory of Mind; t, t statistic

Overall, timecourse responses in the two networks were strikingly similar across groups (see Fig. 9 for group averaged timeseries for each network), however investigating the social brain networks separately suggested differential effects to some degree. For the ToM network, we found reduced within-network inter-regional correlations in the autistic group (see Fig. 4), even though the effect was overall small. Inter-regional correlations were similar between groups within the Pain network, as well as when comparing across regions from both networks. We further observed reduced similarity to the average neurotypical response in autism for both ToM and Pain networks, yet effects remained weak as well. Within- and across-network IRC was correlated with typical network similarity for both groups and both networks. With regard to heterogeneity, network responses were significantly more similar within-group as compared to across-group in neurotypical participants, whereas autistic participants exhibited greater across- than within-group similarity. Within-group similarity was increased in the neurotypical as compared to autistic participants for both networks, further suggesting increased autistic heterogeneity. The relationship between behavioral ToM performance and ToM network connectivity differed between groups, with a positive association only for neurotypical participants.

### Different effects across the social brain networks

With respect to differences in the social brain networks underlying specific inferences about others, we hypothesized to find different connectivity in the ToM and typical connectivity in the Pain network. This dissociation on the neural level was motivated behaviorally by a hypothesis predicting greater difficulty with cognitive inferences about others and less difficulty in empathic concerns for others (“empathy imbalance” [30]) in autistic people. For the ToM network, our findings showing reduced inter-region correlation and reduced similarity to the typical network timecourse response, thus support our hypothesis of alterations in brain network function underlying mental state inferences. Instead of performance-based tasks with explicit instructions, we chose to use a passive movie watching paradigm. This choice was intended to capture more spontaneous (rather than instructed) mental state inferences, thus increasing ecological validity. The observed effects in the ToM network, however, remained weak (Cohen’s *d* between 0.15 and 0.3), which was generally consistent with prior findings on neural responses underlying ToM in ASD [52, 59, 63]. For the Pain network, we found similar correlations amongst the respective network brain regions when comparing the autistic and neurotypical group. This indicates that - at least at the spatiotemporal resolution available - regions responded similarly to events inducing empathic concerns for others’ physical and bodily states across the movie. Interestingly though, when comparing similarity of participants’ individual timecourse response in the Pain network to the average typical timecourse, neurotypical participants were more similar than autistic participants. While this effect was small - it suggests group differences in the precise Pain network responses across time. One possibility could be that the exact timing of empathic responses to certain events in the movie may vary in the autistic participants. An alternative (but not necessarily mutually exclusive) explanation may be that autistic responses in the Pain network are generally more idiosyncratic (when compared to the average neurotypical) across participants, while consistent in the network within an individual. This interpretation would be in line with our findings regarding autistic heterogeneity, as discussed in greater detail below.

To further investigate functional network specialization, we assessed across network inter-region correlations. Against our hypothesis, we did not observe weaker IRC in the autistic group, instead we found similarly strong across-network correlations as compared to the neurotypical group. This result highlights again the relatively typical overall social brain network specialization in adult autistic participants: regions sensitive to thinking about others’ mental states and physical sensations seem to be distinct in their functional response.

### Heterogeneity

Autistic symptomatology can vary significantly across individuals along the larger spectrum, but also across individuals within a smaller range of the autism spectrum (as included in this study). It has been hypothesized that the apparent heterogeneity on the behavioral level is likely reflected by substantial heterogeneity of underlying neural responses [63, 106]. Heterogeneity is often taken to explain small effect sizes or inconsistent findings, and thus needs to be carefully addressed and quantified. We thus tested heterogeneity in several ways in the current study by comparing not only within-group similarity (or dissimilarity) but also potential effects in the relationship for within- versus across-group similarity. First, the analyses revealed greater heterogeneity in the within-group timecourse responses within the autistic as group compared to neurotypical participants. Second, the social brain network responses in autistic participants were less similar to the rest of the autistic group and more similar to the neurotypical group. In contrast, the opposite pattern was evident in the neurotypical group, showing overall more homogenous responses in the ToM and Pain networks.

Greater heterogeneity of neural responses in autistic participants has been previously reported in fMRI timecourse responses. Here, greater idiosyncrasy of responses could reflect increased levels of “noise” across participants (see discussion in [107]). Whether the “noise” is due to actual increased variability, or potentially related to non-BOLD signal variations (e.g., different levels or type of head motion, not adequately captured by the present motion matching) remains to be clarified. With respect to the first possibility, exploring different effects across subgroups (created from either neural or behavioral data) may be informative, however, this was not feasible in the current study due to the small sample size restricted by the COVID-19 pandemic (see discussion in limitation section for future suggestions). Furthermore, some metrics showed non-trivial variability within the NT group (e.g., AQ scores) reflecting individual differences in social cognition that may not be specific to ASD.

Regarding differences in autistic versus neurotypical (i.e., non-autistic) empathy, a “double empathy” [108] challenge has been described. According to this idea, both agents in the interaction have difficulty in understanding each other. In contrast to what we observed, this idea would likely reflect greater similarity of neural responses underlying empathic concern within the groups (as compared to across). Of note, we are not investigating empathy during an actual interaction between the involved agents, whereas the neural basis of empathy imbalance would likely benefit from an interactive design.

### Brain-behavior relationships

We tested the behavioral relevance of the neural findings by directly assessing brain-behavior relationships using preregistered analyses. Specifically, we related neural responses during movie watching to social behavior assessed outside of the scanner via previously established self-report and performance metrics. We observed that the relationship between connectivity within the ToM network and mental state inference behavior using the MASC differed between the groups, although not in the direction of our hypothesis. The neurotypical participants showed a positive correlation (increased ToM behavioral performance related to increased within-ToM IRC) with a small effect size (d = 0.03, *p* = 0.03 not passing conservative multiple comparison correction (alpha = 0.025)), while the autistic participants, contrary to our hypothesis, did not show a significant relationship between the two measures.

To assess whether severity of autistic symptoms may be linked to alterations in social brain networks, we expected ToM network connectivity to be correlated with levels of self-reported autistic symptoms (assessed with the AQ), but did not find a significant relationship. The AQ is a widely used measure for screening and research purposes, but it may not be sensitive enough to capture nuanced differences in social cognition or brain-behavior relationships in our sample. The AQ can be problematic in individuals that lack introspection leading to low scores for otherwise high symptom severity scores, as revealed by metrics not dependent on self-report (e.g., the ADOS). Furthermore, neurotypical participants can also sometimes score above the indicated cut-off (as in the original publication, see [86]). Of note, the neurotypical sample showed some relatively high scores (one even above cut-off). We did not exclude these participants in the full sample, as they did not show otherwise lower socio-cognitive functioning on other metrics. Importantly, levels of AQ in the NT group were low in the closely matched sample, which ultimately replicated the full sample brain-behavior results. It is thus unlikely that the neurotypical participants with relatively high AQ were driving the null result for the AQ.

The choice of socio-cognitive measures might also contribute to the lack of a brain-behavior relationship between performance in the MASC and response magnitude to a previously selected ToM event in the movie (timepoint “T04”, Richardson et al., [54]). Whereas the MASC specifically measures the ability to perform adequate mental state inferences, metrics highlighted by previous studies used the Social Responsiveness Scale (SRS [109]) measuring autistic symptoms more broadly.

Lastly, we tested for a relationship between self-reported overall empathy (measured by the EQ) and within-network connectivity of the Pain network, hypothesizing a positive relationship (higher empathy scores, higher IRC). The fact that we did not observe a significant relationship could have several reasons. For instance, it might imply that the general empathy (including cognitive empathy, emotional empathy and social skills), as assessed with the EQ, is not sufficiently reflecting variability in the Pain network responses. Another possibility would be that similar to the AQ, self-report metrics may not be sufficiently sensitive to capture underlying brain responses (see respective discussion in the *Limitation* section).

### Limitations

Several limitations should be noted. The sample size for the ASD group was relatively small. Disruptions caused by the COVID-19 pandemic limited data acquisition and thus the power to detect group differences, generalization and subgroups assessments. Although we included exploratory analyses using an even more closely matched sample than the full sample, generalizability of the present findings may still be limited by the small sample size. In efforts to improve generalizability, data in this study included two different acquisition sites. While outside of the scope of the current study (see details in the preregistration), future studies may be able to increase sample size by combining samples across studies (e.g., via mega-analyses [110]). While our findings highlight increased heterogeneity in ToM network responses among autistic individuals, our use of a traditional case-control design limits our ability to fully characterize the nature of this variability. Current approaches to studying heterogeneity in autism increasingly rely on subtyping or dimensional frameworks, which may offer more sensitive methods for capturing variation in neural and behavioral phenotypes. Due to the small sample size in the present study, we were underpowered to implement such approaches. Future research with larger sample sizes is needed to explore the observed heterogeneity in neural responses by assessing subgroups that can ultimately more accurately capture the complexities in phenotypes. Importantly, naturalistic paradigms such as movie viewing may be especially well-suited for such approaches, as they can capture rich, temporally dynamic neural responses that may differ meaningfully across subgroups or dimensions within the autistic population.

The current study used passive viewing of social dynamic stimuli not involving explicit instruction to make active inferences about others’ mental or physical bodily states. While limiting direct measures of social behavior during passive movie watching, we consider it a strength to assess spontaneous social information processing and its underlying responses in the brain. Implicit processing — or at least not explicitly instructed (see, e.g [78, 79, 93, 111, 112]), — of dynamic and complex social interactions amongst multiple characters could be more ecologically valid, and thus potentially more diagnostic of the autistic phenotype. To directly relate functional network specialization and similarity to individual behavior within one task, however, future designs should consider combining minimized task instructions with explicit behavioral measurements during neural data acquisition, or shortly thereafter. It is also worth mentioning that the movie used in the study was geared towards a child audience. Social inferences to understand the plot and relationships between the characters are fairly simple. This aspect of the movie may be not complex enough in terms of ToM demands to reveal more subtle difficulty in the autistic group, whereas it has proven powerful to detect functional specialization of the social brain networks in adults (see [97] for a formal comparison to general linear model performance-based study designs).

Only autistic adults were included in this study. As a neurodevelopmental condition with changes in crucial phases during development, the current results are not informative towards mechanisms of developmental trajectories. Autism is, however, also a lifelong condition. Given the increasing number of adults with increasing prevalence, there is thus a great need to understand the “outcome” of the autistic development persisting in adulthood. Characterizing autism in adults is necessary for the development of potential future biomarkers applicable to adults, if neuroimaging is ever to help improve treatment selection or evaluation of individual change in response to treatment.

We also want to note that the generalization of results from neuroimaging studies with autistic participants is always limited to samples that can be ethically included in the study procedures (i.e., who can be comfortable with minimal motion in the fMRI scanner without sedation). Consequently, cognitively more severely affected individuals (e.g., below typical intellectual functioning) or individuals with prominent motor repetitions are typically excluded from participation. Here, passive movie paradigms, using engaging and short films, such as the one included in this study, may in fact hold future promise to extend neuroimaging research to autistic individuals that may otherwise be excluded. This may also help bridge the gap between controlled lab-based tasks and (more) real-world social cognition.

## Conclusions

Functional specialization of the social brain networks underlying inferences about others’ mind and physical states was overall intact (i.e., networks showed specific distinct network responses) in the autistic group. We found small group effects in the neural responses of regions in the ToM network only, and similarity to the average typical response for both networks. Network responses were more idiosyncratic and heterogenous in the autistic group. Brain-behavior relationships differed for ToM behavior only. In sum, we found some, but weak evidence for greater difficulty in the brain networks underlying mental state inferences in autism than empathic concerns, consistent with our preregistered hypotheses. We outline the need and specific suggestions for replicating, generalizing and extending these results in future research.

## Electronic supplementary material

Below is the link to the electronic supplementary material.

Supplementary Material 1

## Data Availability

A preregistration and data are available at https://osf.io/duwk9/. The preregistration outlines the hypotheses and analyses details in this work. Code for fMRI data and statistical analyses are available on public repositories [https://github.com/adolphslab/rsDenoise; https://research-git.uiowa.edu/scnlabp/avp_pc].

## Abbreviations

ADOS: Autism diagnostic observation Schedule
AI: Anterior insula
AMCC: Anterior middle cingulate cortex
AQ: Autism spectrum quotient
ASD: Autism spectrum disorder
EQ: Empathy quotient
FC: Functional connectivity
Fdfilt4: Filtered framewise displacement
FSIQ: Full scale intelligence quotient
IRC: Inter-regional correlation analyses
ISC: Inter-subject correlation analyses
MASC: Movie for the assessment of social cognition
MFG: Medial frontal gyrus
MPFC: Medial prefrontal cortex
MRI: Magnetic resonance imaging
NT: Neurotypical
PC: Precuneus
PDD-NOS: Pervasive developmental disorder-not otherwise specified
ROI: Regions of interest
S2: Somatosensory cortex
SRS: Social responsiveness scale
ToM: Theory of mind
TPJ: Temporoparietal junction
TR: Repetition time
WASI: Wechsler abbreviated scale of intelligence
