# Supplementary material for: Social inference brain networks in autistic adults during movie-viewing: functional specialization and heterogeneity
Source: Mol Autism. 2025 Aug 22;16:42. doi: 10.1186/s13229-025-00669-x (PMC12372314; doi:10.1186/s13229-025-00669-x)
Supplement: Supplementary file 1 — Supplementary Material 1 [file 13229_2025_669_MOESM1_ESM.docx]

**SUPPLEMENTARY INFORMATION**

Clarifications and differences to preregistration

All decisions were made prior to and independent of assessing final group based hypotheses.

- We only specified z-normalization of timeseries data for the magnitude analysis in the pre-registration document. To be consistent across all analyses, all timeseries data was z-normalized.
- Only directly specified for the IRC analyses, all correlation values (r) were fisher r-to-z transformed prior to further calculations.
- We used a butterworth filter during preprocessing, which was previously not specified.
- More in line with Richardson et al., 2018, we excluded the first 13 timepoints (i.e., 9.36 seconds) of the timeseries data
- We reported effect sizes for the statistical analyses.

**Methods**

***MRI Preprocessing Details***

Results included in this manuscript come from preprocessing performed using fMRIPrep 20.0.7 (Esteban, Markiewicz, et al. (2018); Esteban, Blair, et al. (2018); RRID:SCR_016216), which is based on Nipype 1.4.2 (Gorgolewski et al. (2011); Gorgolewski et al. (2018); RRID:SCR_002502).

*Anatomical data processing.* The T1-weighted (T1w) image was corrected for intensity non-uniformity (INU) with N4BiasFieldCorrection (Tustison et al. 2010), distributed with ANTs 2.2.0 (Avants et al. 2008, RRID:SCR_004757), and used as T1w-reference throughout the workflow. The T1w-reference was then skull-stripped with a Nipype implementation of the antsBrainExtraction.sh workflow (from ANTs), using OASIS30ANTs as target template. Brain tissue segmentation of cerebrospinal fluid (CSF), white-matter (WM) and gray-matter (GM) was performed on the brain-extracted T1w using fast (FSL 5.0.9, RRID:SCR_002823, Zhang, Brady, and Smith 2001). Brain surfaces were reconstructed using recon-all (FreeSurfer 6.0.1, RRID:SCR_001847, Dale, Fischl, and Sereno 1999), and the brain mask estimated previously was refined with a custom variation of the method to reconcile ANTs-derived and FreeSurfer-derived segmentations of the cortical gray-matter of Mindboggle (RRID:SCR_002438, Klein et al. 2017). Volume-based spatial normalization to two standard spaces (MNI152NLin6Asym, MNI152Nlin2009cAsym) was performed through nonlinear registration with antsRegistration (ANTs 2.2.0), using brain-extracted versions of both T1w reference and the T1w template. The following templates were selected for spatial normalization: FSL’s MNI ICBM 152 non-linear 6th Generation Asymmetric Average Brain Stereotaxic Registration Model [Evans et al. (2012), RRID:SCR_002823; TemplateFlow ID: MNI152NLin6Asym], ICBM 152 Nonlinear Asymmetrical template version 2009c [Fonov et al. (2009), RRID:SCR_008796; TemplateFlow ID: MNI152NLin2009cAsym].

*Functional data preprocessing.* For each of the available BOLD runs found per subject (across all tasks and sessions), the following preprocessing was performed. First, a reference volume and its skull-stripped version were generated using a custom methodology of fMRIPrep. A B0-nonuniformity map (or fieldmap) was estimated based on two (or more) echo-planar imaging (EPI) references with opposing phase-encoding directions, with 3dQwarp Cox and Hyde (1997) (AFNI 20160207). Based on the estimated susceptibility distortion, a corrected EPI (echo-planar imaging) reference was calculated for a more accurate co-registration with the anatomical reference. The BOLD reference was then co-registered to the T1w reference using bbregister (FreeSurfer) which implements boundary-based registration (Greve and Fischl 2009). Co-registration was configured with six degrees of freedom. Head -motion parameters with respect to the BOLD reference (transformation matrices, and six corresponding rotation and translation parameters) are estimated before any spatiotemporal filtering using mcflirt (FSL 5.0.9, Jenkinson et al. 2002). The BOLD time-series (including slice-timing correction when applied) were resampled onto their original, native space by applying a single, composite transform to correct for head -motion and susceptibility distortions. These resampled BOLD time-series will be referred to as preprocessed BOLD in original space, or just preprocessed BOLD. The BOLD time-series were resampled into standard space, generating a preprocessed BOLD run in MNI152NLin6Asym space. First, a reference volume and its skull-stripped version were generated using a custom methodology of fMRIPrep. Several confounding time-series were calculated based on the preprocessed BOLD: framewise displacement (FD), DVARS and three region-wise global signals. FD and DVARS are calculated for each functional run, both using their implementations in Nipype (following the definitions by Power et al. 2014). The three global signals are extracted within the CSF, the WM, and the whole-brain masks. Additionally, a set of physiological regressors were extracted to allow for component-based noise correction (CompCor, Behzadi et al. 2007). Principal components are estimated after high-pass filtering the preprocessed BOLD time-series (using a discrete cosine filter with 128s cut-oﬀ) for the two CompCor variants: temporal (tCompCor) and anatomical (aCompCor). tCompCor components are then calculated from the top 5% variable voxels within a mask covering the subcortical regions. This subcortical mask is obtained by heavily eroding the brain mask, which ensures it does not include cortical GM regions. For aCompCor, components are calculated within the intersection of the aforementioned mask and the union of CSF and WM masks calculated in T1w space, after their projection to the native space of each functional run (using the inverse BOLD-to-T1w transformation). Components are also calculated separately within the WM and CSF masks. For each CompCor decomposition, the k components with the largest singular values are retained, such that the retained components’ time series are suﬃcient to explain 50 percent of variance across the nuisance mask (CSF, WM, combined, or temporal). The remaining components are dropped from consideration. The head-motion estimates calculated in the correction step were also placed within the corresponding confounds file. The confound time series derived from head motion estimates and global signals were expanded with the inclusion of temporal derivatives and quadratic terms for each (Satterthwaite et al. 2013). Frames that exceeded a threshold of 0.4 mm FD or 1.5 standardised DVARS were annotated as motion outliers. All resamplings can be performed with a single interpolation step by composing all the pertinent transformations (i.e. head-motion transform matrices, susceptibility distortion correction when available, and co-registrations to anatomical and output spaces). Gridded (volumetric) resamplings were performed using antsApplyTransforms (ANTs), configured with Lanczos interpolation to minimize the smoothing eﬀects of other kernels (Lanczos 1964). Non-gridded (surface) resamplings were performed using mri_vol2surf (FreeSurfer). Calculating in-scanner head motion.  Using the head motion parameters derived from fMRIPrep, we computed filtered framewise displacement traces (FDfilt4) as the sums of the backwards diﬀerence of four TRs of motion parameters. The head motion parameters had been filtered to exclude respiratory frequencies (Power et al., 2019). Compared to framewise displacement as computed by fMRIPrep (Power et al., 2019), FDfilt4 is better at separating motion parameters from respiratory fluctuations in multiband acquisitions. Scans with excessive motion were excluded. Excessive motion was identified when mean FDfilt4 is greater than median FDfilt4 plus 1.5 times the IQR of the mean FDfilt4 across all scans. The following exclusion thresholds were calculated for each site: mean FDfilt4 >.4808 for IU, mean FDfilt4 > .5625 for Caltech. The 10 frames before the excessive motion (FDfilt4 > 3.75mm) and 30 frames after were censored.

# **Table S1.** Network connectivity (IRC): ANOVA results and post-hoc linear model coefficients (Full Matched Sample)

|  | **ANOVA** | | | **Model Coefficients** | | | | | |
| --- | --- | --- | --- | --- | --- | --- | --- | --- | --- |
|  | **F** | ***p*** | **Eta^2^** | ***b*** | **B** | **SE** | ***t*** | ***p*** | ***d* [95% CI]** |
| **Linear models with group x site interaction included** (df=1,93) | | | | | | | | | |
| **within-ToM**  Group  mFD  Age  Sex  FSIQ  Site  Group*Site | 3.65  0.37  2.76  0.42  0.51  0.06  1.47 | 0.06  0.55  0.10  0.52  0.48  0.81  0.23 | 0.04  0.004  0.03  0.004  0.005  0.004  0.01 | 0.06  -0.07  -0.002  -0.01  -0.0005  0.04  -0.06 | 0.27  -0.06  -0.16  -0.04  -0.05  0.20  -0.24 | 0.03  0.13  0.002  0.02  0.001  0.04  0.05 | 2.14  -0.57  -1.32  -0.38  -0.49  1.07  -1.21 | 0.04  0.57  0.19  0.70  0.63  0.29  0.23 | 0.21 [-0.20, 0.61]  -0.05 [-0.46, 0.35]  -0.13 [-0.53, 0.28]  -0.04 [-0.44, 0.37]  -0.05 [-0.45, 0.36]  0.10 [-0.30, 0.51]  -0.12 [-0.52, 0.29] |
| **within-Pain**  Group  mFD  Age  Sex  FSIQ  Site  Group*Site | 0.26  0.21  5.66  1.20  1.60  0.07  0.72 | 0.61  0.65  0.02  0.28  0.21  0.79  0.40 | 0.003  0.002  0.06  0.01  0.02  0.001  0.01 | 0.02  -0.07  -0.003  -0.02  -0.001  0.02  -0.04 | 0.10  -0.05  -0.21  -0.09  -0.13  0.09  -0.17 | 0.03  0.13  0.002  0.02  0.001  0.04  0.05 | 0.78  -0.51  -1.79  -0.91  -1.20  0.46  -0.85 | 0.44  0.61  0.08  0.37  0.23  0.65  0.40 | 0.08 [-0.33, 0.48]  -0.05 [-0.46, 0.36]  -0.17 [-0.58, 0.24]  -0.09 [-0.49, 0.32]  -0.12 [-0.52, 0.29]  0.04 [-0.36, 0.45]  -0.08 [-0.49, 0.33] |
| **across**  Group  mFD  Age  Sex  FSIQ  Site  Group*Site | 1.24  16.08  0.59  0.31  2.87  0.66  1.33 | 0.27  0.0001  0.44  0.58  0.09  0.42  0.25 | 0.01  0.14  0.01  0.002  0.02  0.01  0.01 | -0.04  0.44  0.001  0.01  -0.002  -0.06  0.05 | -0.16  0.35  0.07  0.03  -0.20  -0.25  0.21 | 0.03  0.12  0.002  0.02  0.001  0.04  0.05 | -1.32  3.66  0.60  0.33  -1.96  -1.40  1.15 | 0.19  0.0004  0.55  0.74  0.05  0.16  0.25 | -0.13 [-0.53, 0.28]  0.36 [-0.05, 0.77]  0.06 [-0.35, 0.46]  0.03 [-0.38, 0.44]  -0.19 [-0.60, 0.22]  -0.14 [-0.54, 0.27]  0.11 [-0.30, 0.52] |
| **Linear models with non-significant group x site interaction removed** (df=1,94) | | | | | | | | | |
| *within-ToM*  Group  mFD  Age  Sex  FSIQ  Site | 3.63  0.36  2.74  0.42  0.51  0.06 | 0.06  0.55  0.10  0.52  0.48  0.81 | 0.04  0.004  0.03  0.004  0.005  0.0005 | 0.04  -0.08  -0.003  -0.01  -0.001  0.01 | 0.18  -0.06  -0.19  -0.06  -0.06  0.03 | 0.02  0.13  0.002  0.02  0.001  0.03 | 1.76  -0.62  -1.60  -0.61  -0.59  0.24 | 0.08  0.54  0.11  0.55  0.55  0.81 | 0.17 [-0.24, 0.58]  -0.06 [-0.47, 0.35]  -0.15 [-0.56, 0.25]  -0.06 [-0.47, 0.35]  -0.06 [-0.46, 0.35]  0.02 [-0.38, 0.43] |
| *within-Pain*  Group  mFD  Age  Sex  FSIQ  Site | 0.26  0.21  5.68  1.21  1.60  0.07 | 0.61  0.65  0.02  0.27  0.21  0.79 | 0.003  0.002  0.06  0.01  0.02  0.0007 | 0.01  -0.07  -0.004  -0.02  -0.001  -0.01 | 0.03  -0.06  -0.24  -0.11  -0.14  -0.03 | 0.02  0.13  0.002  0.02  0.001  0.03 | 0.34  -0.55  -2.01  -1.08  -1.28  -0.27 | 0.73  0.58  0.047  0.28  0.20  0.79 | 0.03 [-0.37, 0.44]  -0.05 [-0.46, 0.35]  -0.19 [-0.60, 0.21]  -0.10 [-0.51, 0.30]  -0.12 [-0.53, 0.28]  -0.03 [-0.43, 0.38] |
| *across*  Group  mFD  Age  Sex  FSIQ  Site | 1.23  16.03  0.59  0.30  2.86  0.66 | 0.27  0.0001  0.45  0.58  0.09  0.42 | 0.01  0.14  0.005  0.003  0.02  0.006 | -0.02  0.45  0.001  0.01  -0.002  -0.02 | -0.07  0.35  0.09  0.05  -0.19  -0.09 | 0.02  0.12  0.002  0.02  0.001  0.03 | -0.78  3.70  0.86  0.54  -1.87  -0.81 | 0.43  0.0004  0.39  0.59  0.07  0.42 | -0.08 [-0.48, 0.33]  0.36 [-0.05, 0.77]  0.08 [-0.32, 0.49]  0.05 [-0.35, 0.46]  -0.18 [-0.59, 0.23]  -0.08 [-0.49, 0.33] |

*Abbreviations:* b, unstandardized beta, B, standardized beta, CI, Confidence Interval, *d*, Cohen’s d, eta^2^, partial eta squared, F, F value, FSIQ, Full Scale Intelligence Quotient, IRC, Inter-region correlations, mFD, mean framewise displacement for all timepoints, *p,* p value, SE, standard error, t, t value, z, Fisher Z-transformed

# **Table S2.** Typical response similarity (ISC): ANOVA results and post-hoc linear model coefficients (Full Matched Sample)

|  | ANOVA | | | Model Coefficients | | | | | |
| --- | --- | --- | --- | --- | --- | --- | --- | --- | --- |
|  | F | *p* | Eta^2^ | *b* | B | SE | *t* | *p* | *d* [95% CI] |
| Linear models with group x site interaction included (df=1,93) | | | | | | | | | |
| ToM  Group  mFD  Age  Sex  FSIQ  Site  Group*Site | 5.00  9.28  9.39  0.99  1.69  0.001  1.32 | 0.03  0.003  0.003  0.32  0.20  0.97  0.25 | 4.2-02  7.7e-02  7. 8e-02  8.2e-03  1.4e-02  1.2e-05  1.1e-02 | 0.10  -0.58  -0.01  0.04  0.002  0.06  -0.09 | 0.27  -0.26  -0.24  0.11  0.13  0.15  -0.21 | 0.04  0.20  0.003  0.04  0.002  0.07  0.08 | 2.32  -2.83  -2.16  1.14  1.33  0.89  -1.15 | 0.02  0.01  0.03  0.26  0.19  0.38  0.25 | 0.22 [-0.18, 0.63]  -0.27 [-0.68, 0.13]  -0.21 [-0.62, 0.20]  0.11 [-0.30, 0.52]  0.13 [-0.28, 0.54]  0.09 [-0.32, 0.49]  -0.11 [-0.52, 0.30] |
| Pain  Group  mFD  Age  Sex  FSIQ  Site  GroupSite | 5.08  13.40  3.46  0.03  0.45  0.08  0.01 | 0.03  0.0004  0.07  0.86  0.50  0.78  0.92 | 4.4e-02  1.2e-01  2.9e-02  2.6e-04  3.9e-03  6.6e-04  9.3e-05 | 0.06  -0.69  -0.004  0.01  0.001  -0.01  -0.01 | 0.18  -0.34  -0.15  0.01  0.06  -0.02  -0.02 | 0.04  0.20  0.003  0.03  0.001  0.06  0.07 | 1.51  -3.56  -1.29  0.15  0.55  -0.11  -0.10 | 0.13  0.001  0.20  0.88  0.58  0.92  0.92 | 0.15 [-0.26, 0.55]  -0.35 [-0.76, 0.06]  -0.12 [-0.53, 0.28]  0.01 [-0.39, 0.42]  0.05 [-0.35, 0.46]  -0.01 [-0.42, 0.40]  -0.01 [-0.42, 0.40] |
| Linear models with non-significant group x site interaction removed (df=1,94) | | | | | | | | | |
| ToM  Group  mFD  Age  Sex  FSIQ  Site | 4.98  9.25  9.36  0.99  1.69  0.001 | 0.03  0.003  0.003  0.32  0.20  0.97 | 4.2e-02  7.7e-02  7.8e-02  8.2e-03  1.4e-02  1.2e-05 | 0.07  -0.59  -0.01  0.03  0.002  0.002 | 0.19  -0.27  -0.27  0.09  0.12  0.004 | 0.04  0.20  0.003  0.04  0.002  0.04 | 2.03  -2.88  -2.45  0.95  1.24  0.04 | 0.04  0.005  0.02  0.34  0.22  0.97 | 0.20 [-0.21, 0.60]  -0.28 [-0.69, 0.13]  -0.24 [-0.64, 0.17]  0.09 [-0.32, 0.50]  0.12 [-0.29, 0.53]  0.004 [-0.40, 0.41] |
| Pain  Group  mFD  Age  Sex  FSIQ  Site | 5.14  13.54  3.50  0.03  0.46  0.08 | 0.03  0.0004  0.06  0.86  0.50  0.78 | 0.04  0.12  0.03  0.0003  0.004  0.001 | 0.06  -0.69  -0.004  0.004  0.001  -0.01 | 0.17  -0.34  -0.15  0.01  0.05  -0.03 | 0.03  0.19  0.003  0.03  0.001  0.04 | 1.82  -3.59  -1.35  0.13  0.55  -0.28 | 0.07  0.001  0.18  0.90  0.59  0.78 | 0.18 [-0.23, 0.58]  0.03 [-0.37, 0.44]  0.13 [-0.28, 0.54]  0.01 [-0.39, 0.42]  0.05 [-0.35, 0.46]  -0.03 [-0.43, 0.38] |

*Abbreviations:* b, unstandardized beta, B, standardized beta, CI, Confidence Interval, *d*, Cohen’s d, eta^2^, partial eta squared, F, F value, FSIQ, Full Scale Intelligence Quotient, ISC, Inter-subject correlations, mFD, mean framewise displacement for all timepoints, *p,* p value, SE, standard error, t, t value, z, Fisher Z-transformed

# **Table S3.** Associations between typical response similarity (ISC) and network connectivity (IRC): ANOVA results and post-hoc linear model coefficients (Full Matched Sample)

|  | ANOVA | | | Model Coefficients | | | | | |
| --- | --- | --- | --- | --- | --- | --- | --- | --- | --- |
|  | F | *p* | Eta^2^ | *b* | B | SE | *t* | *p* | *d* [95% CI] |
| Linear models with group interactions included (df=1,89) | | | | | | | | | |
| ToM  across  within_ToM  Group  mFD  Age  Sex  FSIQ  Site  Group* Site  across*Group  within_ToM*Group | 44.16  15.61  1.19  0.41  6.31  2.92  0.47  0.29  0.01  1.02  1.53 | 2.4e-09  0.0002  0.28  0.52  0.01  0.09  0.50  0.59  0.93  0.32  0.22 | 0.27  0.10  0.01  0.003  0.04  0.02  0.003  0.002  0.00005  0.01  0.01 | -1.20  0.78  0.18  -0.14  -0.004  0.05  0.001  -0.04  0.01  0.42  -0.43 | -0.71  0.45  0.47  -0.07  -0.16  0.13  0.05  -0.10  0.01  0.22  -0.54 | 0.27  0.30  0.14  0.19  0.003  0.03  0.001  0.06  0.07  0.32  0.35 | -4.41  2.57  1.26  -0.75  -1.65  1.61  0.60  -0.60  0.09  1.32  -1.24 | 2.9e-05  0.01  0.21  0.46  0.10  0.11  0.55  0.55  0.93  0.19  0.22 | -0.43 [-0.84, -0.02]  0.25 [-0.16, 0.66]  0.12 [-0.29, 0.53]  -0.07 [-0.48, 0.34]  -0.16 [-0.57, 0.25]  0.16 [-0.25, 0.56]  0.06 [-0.35, 0.46]  -0.06 [-0.47, 0.35]  0.01 [-0.40, 0.42]  0.13 [-0.28, 0.54]  -0.12 [-0.53, 0.29] |
| Pain  across  within_Pain  Group  mFD  Age  Sex  FSIQ  Site  Group*Site  across*Group  within_Pain*Group | 16.13  17.02  2.81  4.12  0.74  0.61  0.30  0.25  0.75  1.91  0.33 | 0.0001  8.3e-05  0.10  0.05  0.39  0.44  0.59  0.62  0.39  0.17  0.57 | 0.12  0.13  0.02  0.03  0.01  0.005  0.002  0.002  0.01  0.01  0.002 | -0.96  0.69  0.07  -0.41  -0.001  0.02  0.0003  -0.07  0.06  0.54  -0.21 | -0.61  0.43  0.18  -0.20  -0.06  0.06  0.02  -0.20  0.15  0.30  -0.27 | 0.30  0.28  0.13  0.20  0.003  0.03  0.001  0.06  0.07  0.34  0.33 | -3.25  2.46  0.50  -2.09  -0.54  0.66  0.20  -1.14  0.87  1.61  -0.65 | 0.002  0.02  0.62  0.04  0.59  0.51  0.84  0.26  0.39  0.11  0.52 | -0.32 [-0.72, 0.09]  0.24 [-0.17, 0.65]  0.05 [-0.36, 0.46]  -0.20 [-0.61, 0.21]  -0.05 [-0.46, 0.36]  0.06 [-0.34, 0.47]  0.02 [-0.39, 0.43]  -0.11 [-0.52, 0.30]  0.08 [-0.32, 0.49]  0.16 [-0.25, 0.56]  -0.06 [-0.47, 0.34] |
| Linear models with non-significant group interactions removed (df=1,93) | | | | | | | | | |
| ToM  across  within_ToM  mFD  Age  Sex  FSIQ  Site | 44.09  15.59  0.53  5.81  2.99  0.26  0.40 | 2.1e-09  0.0002  0.47  0.02  0.09  0.61  0.40 | 0.27  0.10  0.003  0.04  0.02  0.002  0.002 | -0.93  0.49  -0.16  -0.004  0.05  0.0004  -0.02 | -0.54  0.29  -0.08  -0.15  0.13  0.02  -0.06 | 0.15  0.14  0.19  0.003  0.03  0.001  0.04 | -6.04  3.41  -0.87  -1.62  1.66  0.27  -0.63 | 3.17e-08  0.001  0.39  0.11  0.10  0.79  0.53 | -0.60 [-1.02, -0.19]  0.33 [-0.08, 0.74]  -0.08 [-0.49, 0.32]  -0.16 [-0.56, 0.25]  0.16 [-0.25, 0.57]  0.03 [-0.38, 0.43]  -0.06 [-0.47, 0.35] |
| Pain  across  within_Pain  mFD  Age  Sex  FSIQ  Site | 15.91  16.79  4.77  0.57  0.62  0.11  0.36 | 0.0001  8.9e-05  0.03  0.45  0.43  0.74  0.55 | 0.12  0.13  0.04  0.004  0.005  0.001  0.003 | -0.58  0.52  -0.44  -0.001  0.02  0.0002  -0.02 | -0.36  0.33  -0.21  -0.03  0.07  0.01  -0.07 | 0.16  0.16  0.20  0.003  0.03  0.001  0.04 | -3.51  3.35  -2.23  -0.27  0.74  0.11  -0.60 | 0.001  0.001  0.03  0.79  0.46  0.91  0.55 | -0.34 [-0.75, 0.07]  0.33 [-0.08, 0.74]  -0.22 [-0.62, 0.19]  -0.03 [-0.43, 0.38]  0.07 [-0.34, 0.48]  0.01 [-0.40, 0.42]  -0.06 [-0.46, 0.35] |

*Abbreviations:* b, unstandardized beta, B, standardized beta, CI, Confidence Interval, *d*, Cohen’s d, eta^2^, partial eta squared, F, F value, FSIQ, Full Scale Intelligence Quotient, IRC, Inter-region correlations, ISC, Inter-subject correlations, mFD, mean framewise displacement for all timepoints, *p,* p value, SE, standard error, t, t value, z, Fisher Z-transformed

# **Table S4.** Brain-behavior relationships: ANOVA results and post-hoc linear model coefficients (Full Matched Sample)

|  | ANOVA | | | Model Coefficients | | | | | |
| --- | --- | --- | --- | --- | --- | --- | --- | --- | --- |
|  | F | *p* | Eta^2^ | *b* | B | SE | *t* | *p* | *d* [95% CI] |
| Linear models with group interactions included | | | | | | | | | |
| within-ToM IRC * MASC (df=81) | | | | | | | | | |
| MASC Score  Group  MASC * group  mFD  Age  Sex  FSIQ  Site  Group * Site | 0.37  1.84  4.95  0.34  1.57  0.83  0.33  0.08  0.56 | 0.54  0.18  0.03  0.56  0.21  0.36  0.57  0.78  0.46 | 0.004  0.02  0.05  0.004  0.02  0.01  0.004  0.001  0.01 | -0.002  -0.28  0.01  -0.06  -0.002  -0.03  -0.0003  0.03  -0.04 | -0.10  -1.22  1.51  -0.04  -0.12  -0.14  -0.04  0.13  -0.17 | 0.003  0.17  0.005  0.14  0.002  0.03  0.001  0.05  0.05 | -0.67  -1.65  2.01  -0.40  -0.95  -1.24  -0.31  0.58  -0.75 | 0.51  0.10  0.05  0.69  0.35  0.22  0.75  0.56  0.46 | -0.07 [-0.52, 0.38]  -0.17 [-0.62, 0.28]  0.21 [-0.24, 0.66]  -0.04 [-0.49, 0.41]  -0.10 [-0.55, 0.35]  -0.13 [-0.58, 0.32]  -0.03 [-0.48, 0.42]  0.06 [-0.39, 0.51]  -0.08 [-0.53, 0.37] |
| within-ToM IRC * AQ (df=91) | | | | | | | | | |
| AQ Score  Group  AQ * group  mFD  Age  Sex  FSIQ  Site  Group * Site | 0.21  3.91  0.35  0.24  2.44  0.39  0.53  0.08  1.31 | 0.65  0.05  0.55  0.62  0.12  0.53  0.47  0.77  0.26 | 0.002  0.04  0.004  0.002  0.02  0.004  0.005  0.001  0.01 | -0.0003  0.03  0.002  -0.07  -0.002  -0.01  -0.001  0.04  -0.06 | -0.03  0.13  0.15  -0.05  -0.14  -0.02  -0.06  0.20  -0.23 | 0.002  0.08  0.003  0.13  0.002  0.02  0.001  0.04  0.05 | -0.15  0.38  0.52  -0.51  -1.13  -0.22  -0.522  1.04  -1.15 | 0.88  0.71  0.61  0.61  0.26  0.83  0.60  0.30  0.26 | -0.02 [-0.43, 0.40]  0.04 [-0.38, 0.45]  -0.05 [-0.36, 0.47]  -0.05 [-0.47, 0.37]  -0.11 [-0.53, 0.30]  -0.02 [-0.44, 0.39]  -0.05 [-0.47, 0.36]  0.10 [-0.31, 0.52]  -0.11 [-0.53, 0.30] |
| T04 peak * MASC (df=80) | | | | | | | | | |
| MASC Score  Group  MASC * group  mFD  Age  Sex  FSIQ  Site  Group * Site | 0.0004  2.88  0.06  1.79  0.26  1.5  1.12  0.68  0.08 | 0.98  0.09  0.81  0.18  0.61  0.22  0.29  0.41  0.77 | 4.5e-06  3.3e-02  6.9e-04  2e-02  2.9e-03  1.7e-02  1.3e-02  7.7e-03  9.4e-04 | -0.005  -0.33  0.005  -0.59  0.001  0.09  0.003  -0.12  0.05 | -0.07  -0.44  0.25  -0.14  0.02  0.12  0.11  -0.17  0.07 | 0.01  0.59  0.02  0.47  0.01  0.09  0.004  0.17  0.19 | -0.43  -0.56  0.32  -1.25  0.14  1.04  0.90  -0.74  0.29 | .67  0.58  0.75  0.21  0.89  0.30  0.37  0.46  0.77 | -0.04 [-0.50, 0.41]  -0.06, [-0.51, 0.40]  0.03 [-0.42, 0.49]  -0.13 [-0.59, 0.32]  0.01 [-0.44, 0.47]  0.11 [-0.35, 0.57]  0.09 [-0.36, 0.55]  -0.08 [-0.53, 0.38]  0.03 [-0.43, 0.49] |
| within-Pain IRC * EQ (df=90) | | | | | | | | | |
| EQ Score  Group  EQ * group  mFD  Age  Sex  FSIQ  Site  Group * Site | 0.94  0.95  0.96  0.12  4.71  0.87  1.61  0.02  0.52 | 0.33  0.33  0.33  0.72  0.03  0.35  0.21  0.88  0.47 | 0.01  0.01  0.01  0.001  0.05  0.01  0.02  0.0002  0.01 | -0.001  -0.04  0.002  -0.08  -0.004  -0.03  -0.001  0.01  -0.04 | -0.17  -0.16  0.36  -0.06  -0.25  -0.11  -0.14  0.06  -0.15 | 0.001  0.07  0.002  0.13  0.002  0.03  0.001  0.04  0.05 | -0.96  -0.49  0.93  -0.63  -1.94  -1.02  -1.25  0.33  -0.72 | 0.34  0.62  0.35  0.53  0.06  0.31  0.21  0.74  0.47 | -0.10 [-0.52, 0.32]  -0.05 [-0.47, 0.37]  0.09 [-0.33, 0.51]  -0.06 [-0.48, 0.36]  -0.19 [-0.62, 0.23]  -0.10 [-0.52, 0.32]  -0.12 [-0.55, 0.30]  0.03 [-0.39, 0.45]  -0.07 [-0.49, 0.35] |
| Linear models with non-significant group interactions removed | | | | | | | | | |
| within-ToM IRC * MASC (df=82) | | | | | | | | | |
| MASC Score  Group  MASC*group  mFD  Age  Sex  FSIQ  Site | 0.38  1.85  4.98  0.34  1.57  0.84  0.33  0.08 | 0.54  0.18  0.03  0.56  0.21  0.36  0.57  0.78 | 0.004  0.02  0.05  0.004  0.02  0.01  0.004  0.001 | -0.002  -0.32  0.01  -0.06  -0.002  -0.04  -0.0003  -0.0005 | -0.14  -1.39  1.63  -0.05  -0.14  -0.16  -0.03  -0.002 | 0.003  0.16  0.005  0.14  0.002  0.02  0.001  0.03 | -0.97  -2.00  2.23  -0.47  -1.08  -1.44  -0.29  -0.02 | 0.33  0.05  0.03  0.64  0.28  0.15  0.77  0.99 | -0.10 [-0.55, 0.35]  -0.21 [-0.66, 0.24]  0.23 [-0.22, 0.69]  -0.05 [-0.50, 0.40]  -0.11 [-0.56, 0.34]  -0.15 [-0.60, 0.30]  -0.03 [-0.48, 0.42]  -0.002 [-0.45, 0.45] |
| within-ToM IRC * AQ (df=94) | | | | | | | | | |
| AQ Score  mFD  Age  Sex  FSIQ  Site | 0.21  1.15  2.70  0.60  0.62  0.0008 | 0.65  0.29  0.10  0.44  0.43  0.98 | 2.1e-03  1.2e-02  2.7e-02  6.1e-03  6.3e-03  7.6e-06 | -0.001  -0.30  -0.003  -0.02  -0.001  -0.001 | -0.07  -0.11  -0.18  -0.08  -0.08  -0.004 | 0.001  0.27  0.002  0.023  0.001  0.03 | -0.71  -1.10  -1.46  -0.75  -0.76  -0.03 | 0.48  0.27  0.15  0.46  0.45  0.98 | -0.07 [-0.49, 0.35]  -0.11 [-0.53, 0.31]  -0.15 [-0.56, 0.27]  -0.07 [-0.49, 0.34]  -0.07 [-0.49, 0.34]  -0.003 [-0.42, 0.41] |
| T04 peak * MASC (df=83) | | | | | | | | | |
| MASC Score  mFD  Age  Sex  FSIQ  Site | 0.0004  1.30  0.44  1.80  2.08  0.53 | 0.98  0.26  0.51  0.18  0.15  0.47 | 4.5e-06  1.5e-02  4.9e-03  2e-02  2.3e-02  5.9e-03 | -0.01  -0.49  0.0003  0.11  0.004  -0.07 | -0.11  -0.11  0.01  0.14  0.15  -0.10 | 0.01  0.46  0.01  0.08  0.004  0.10 | -0.96  -1.06  0.04  1.30  1.26  -0.73 | 0.34  0.29  0.97  0.20  0.21  0.47 | -0.1 [-0.56, 0.36]  -0.11 [-0.57, 0.35]  0.005 [-0.45, 0.46]  0.14 [-0.32, 0.59]  0.13 [-0.32, 0.59]  -0.08 [-0.53, 0.38] |
| within-Pain IRC * EQ (df=93) | | | | | | | | | |
| EQ Score  mFD  Age  Sex  FSIQ  Site | 0.95  0.26  4.92  1.02  1.80  0.05 | 0.33  0.61  0.03  0.32  0.18  0.83 | 0.009  0.003  0.05  0.01  0.02  0.0005 | -0.0002  -0.08  -0.004  -0.02  -0.001  -0.01 | -0.04  -0.06  -0.23  -0.10  -0.14  -0.03 | 0.001  0.13  0.002  0.02  0.001  0.03 | -0.36  -0.63  -1.92  -0.96  -1.34  -0.22 | 0.72  0.53  0.06  0.34  0.18  0.83 | -0.04 [-0.46, 0.38]  -0.06 [-0.48, 0.36]  -0.19 [-0.61, 0.23]  -0.10 [-0.52, 0.33]  -0.13 [-0.55, 0.29]  -0.02 [-0.44, 0.40] |

*Abbreviations:* AQ, Autism Spectrum Quotient, b, unstandardized beta, B, standardized beta, CI, Confidence Interval, *d*, Cohen’s d, eta^2^, partial eta squared, EQ, Empathy Quotient, F, F value, FSIQ, Full Scale Intelligence Quotient, IRC, Inter-region correlations, MASC, Movie for the Assessment of Social Cognition, mFD, mean framewise displacement for all timepoints, *p,* p value, SE, standard error, t, t value, z, Fisher Z-transformed

**Table S5.** Network connectivity (IRC): ANOVA results and post-hoc linear model coefficients (Matched Sample)

|  | ANOVA | | | Model Coefficients | | | | | |
| --- | --- | --- | --- | --- | --- | --- | --- | --- | --- |
| Model | F | *p* | Eta^2^ | *b* | B | SE | *t* | *p* | *d* [95% CI] |
| within-ToM IRC (df=60) | | | | | | | | | |
| Group  mFD  Age  Sex  FSIQ  Site | 4.54  2.24  1.97  0.15  1.20  0.89 | 0.04  0.14  0.17  0.70  0.28  0.35 | 0.06  0.03  0.03  0.002  0.02  0.01 | 0.05  -0.24  -0.004  -0.01  -0.001  0.03 | 0.26  -0.19  -0.25  -0.03  -0.08  0.15 | 0.02  0.15  0.003  0.03  0.001  0.03 | 2.17  -1.60  -1.72  -0.24  -0.61  0.95 | 0.03  0.11  0.09  0.81  0.55  0.35 | 0.27 [-0.22, 0.75]  -0.20 [-0.68, 0.29]  -0.21 [-0.69, 0.27]  -0.03 [-0.51, 0.45]  -0.07 [-0.55, 0.41]  0.11 [-0.36, 0.59] |
| within-ToM IRC (df=60) | | | | | | | | | |
| Group  mFD  Age  Sex  FSIQ  Site | 0.03  2.14  2.91  0.71  2.83  0.001 | 0.86  0.15  0.09  0.40  0.10  0.97 | 4.7e-04  3.1e-02  4.2e-02  1e-02  4.1e-02  1.8e-05 | 0.01  -0.28  -0.004  -0.02  -0.002  0.001 | 0.03  -0.22  -0.22  -0.10  -0.20  0.01 | 0.03  0.16  0.003  0.03  0.001  0.03 | 0.25  -1.75  -1.48  -0.80  -1.52  0.04 | 0.80  0.08  0.14  0.43  0.13  0.97 | 0.03 [-0.45, 0.51]  -0.21 [-0.69, 0.27]  -0.18 [-0.66, 0.30]  -0.10 [-0.58, 0.38]  -0.19 [-0.66, 0.30]  0.004 [-0.47, 0.48] |
| across-network IRC (df=60) | | | | | | | | | |
| Group  mFD  Age  Sex  FSIQ  Site | 0.06  2.93  0.22  0.21  1.51  0.71 | 0.82  0.09  0.64  0.65  0.22  0.40 | 0.001  0.04  0.003  0.003  0.02  0.01 | -8.2e-03  2e-01  2.5e-05  9.1e-03  -1.5e-03  -2.6e-02 | -4.31-02  1.7e-01  1.5e-03  4.4e-02  -2e-01  -1.43e-01 | 2.40-02  1.5e-01  2.5e-03  2.6e-02  1.1e-03  3.1e-02 | -0.34  1.37  0.01  0.36  -1.47  -0.84 | 0.73  0.17  0.99  0.72  0.15  0.40 | -0.04 [-0.52, 0.44]  0.17 [-0.31, 0.65]  0.001 [-0.48, 0.48]  0.04 [-0.44, 0.52]  -0.18 [-0.66, 0.30]  -0.10 [-0.58, 0.38] |

*Abbreviations:* b, unstandardized beta, B, standardized beta, CI, Confidence Interval, *d*, Cohen’s d, eta^2^, partial eta squared, F, F value, FSIQ, Full Scale Intelligence Quotient, IRC, Inter-region correlations, mFD, mean framewise displacement for all timepoints, *p,* p value, SE, standard error, t, t value, z, Fisher Z-transformed

**Table S6.** Typical response similarity (ISC): ANOVA results and post-hoc linear model coefficients (Matched Sample)

|  | ANOVA | | | Model Coefficients | | | | | |
| --- | --- | --- | --- | --- | --- | --- | --- | --- | --- |
| Model | F | *p* | Eta^2^ | *b* | B | SE | *t* | *p* | *d* [95% CI] |
| ToM typical response similarity (df=60) | | | | | | | | | |
| Group  mFD  Age  Sex  FSIQ  Site | 2.63  7.42  1.81  3.26  0.0001  0.26 | 0.11  0.008  0.18  0.08  0.99  0.61 | 3.5e-02  9.9e-02  2.4e-02  4.3e-02  1.3e-06  3.4e-03 | 0.08  -0.74  -0.01  0.08  0.0004  0.03 | 0.21  -0.33  -0.20  0.22  0.03  0.08 | 0.04  0.26  0.004  0.05  0.002  0.06 | 1.80  -2.82  -1.45  1.86  0.22  0.51 | 0.08  0.01  0.15  0.07  0.83  0.61 | 0.22 [-0.26, 0.70]  -0.35 [-0.83, 0.14]  -0.18 [-0.66, 0.30]  0.23 [-0.25, 0.71]  0.03 [-0.45, 0.51]  0.06 [-0.42, 0.54] |
| Pain typical response similarity (df=60) | | | | | | | | | |
| Group  mFD  Age  Sex  FSIQ  Site | 3.32  10.77  2.44  0.10  0.14  0.002 | 0.07  0.002  0.12  0.75  0.71  0.97 | 4.3e-02  1.4e-01  3.2e-02  1.3e-03  1.9e-03  2.2e-05 | 0.07  -0.75  -0.005  0.01  -0.001  -0.002 | 0.24  -0.40  -0.18  0.04  -0.05  -0.01 | 0.04  0.22  0.004  0.04  0.002  0.05 | 2.02  -3.43  -1.31  0.31  -0.36  -0.04 | 0.05  0.001  0.20  0.76  0.72  0.97 | 0.25 [-0.23, 0.73]  -0.43 [-0.91, 0.06]  -0.16 [-0.64, 0.32]  0.04 [-0.44, 0.52]  -0.04 [-0.52, 0.44]  -0.01 [-0.48, 0.47] |

*Abbreviations:* b, unstandardized beta, B, standardized beta, CI, Confidence Interval, *d*, Cohen’s d, eta^2^, partial eta squared, F, F value, FSIQ, Full Scale Intelligence Quotient, ISC, Inter-subject correlations, mFD, mean framewise displacement for all timepoints, *p,* p value, SE, standard error, t, t value, z, Fisher Z-transformed

**Table S7.** Associations between typical response similarity (ISC) and network connectivity (IRC): ANOVA results and post-hoc linear model coefficients (Matched Sample)

|  | ANOVA | | | Model Coefficients | | | | | |
| --- | --- | --- | --- | --- | --- | --- | --- | --- | --- |
| Model | F | *p* | Eta^2^ | *b* | B | SE | *t* | *p* | *d* [95% CI] |
| ToM typical response similarity ~ IRC (df=59) | | | | | | | | | |
| across_IRC  within_IRC  mFD  Age  Sex  FSIQ  Site | 34.41  14.30  1.68  1.75  8.10  0.53  0.23 | 2.156e-07  0.0004  0.20  0.19  0.006  0.47  0.63 | 0.29  0.12  0.01  0.01  0.07  0.004  0.002 | -1.24  0.61  -0.34  -0.003  0.10  -0.001  -0.02 | -0.65  0.33  -0.15  -0.11  0.26  -0.09  -0.06 | 0.19  0.19  0.22  0.004  0.04  0.001  0.05 | -6.37  3.26  -1.56  -0.94  2.78  -0.86  -0.48 | 3.18e-08  0.002  0.12  0.35  0.007  0.39  0.63 | -0.84 [-1.34, -0.34]  0.40 [-0.08, 0.89]  -0.19 [-0.67, 0.29]  -0.11 [-0.59, 0.36]  0.34 [-0.14, 0.82]  -0.10 [-0.58, 0.37]  -0.06 [-0.54, 0.42] |
| Pain typical response similarity ~ IRC (df=59) | | | | | | | | | |
| across_IRC  within_IRC  mFD  Age  Sex  FSIQ  Site | 12.80  20.21  3.13  0.75  1.19  0.35  0.31 | 0.0007  3.297e-05  0.08  0.39  0.28  0.56  0.58 | 0.13  0.21  0.03  0.01  0.01  0.004  0.003 | -0.83  0.62  -0.40  -0.002  0.03  -0.001  -0.02 | -0.51  0.43  -0.22  -0.06  0.10  -0.09  -0.07 | 0.19  0.17  0.21  0.003  0.03  0.001  0.04 | -4.39  3.55  -1.95  -0.48  1.01  -0.77  -0.55 | 4.81e-05  0.001  0.06  0.63  0.31  0.45  0.58 | -0.55 [-1.04, -0.06]  0.44 [-0.04, 0.93]  -0.24 [-0.72, 0.24]  -0.06 [-0.54, 0.42]  0.12 [-0.36, 0.60]  -0.09 [-0.57, 0.39]  -0.07 [-0.55, 0.41] |

*Abbreviations:* b, unstandardized beta, B, standardized beta, CI, Confidence Interval, *d*, Cohen’s d, eta^2^, partial eta squared, F, F value, FSIQ, Full Scale Intelligence Quotient, IRC, Inter-region correlations, ISC, Inter-subject correlations, mFD, mean framewise displacement for all timepoints, *p,* p value, SE, standard error, t, t value, z, Fisher Z-transformed

**Table S8.** Brain-behavior relationships: ANOVA results and post-hoc linear model coefficients (Matched Sample)

|  | ANOVA | | | Model Coefficients | | | | | |
| --- | --- | --- | --- | --- | --- | --- | --- | --- | --- |
| Model | F | *p* | Eta^2^ | *b* | B | SE | *t* | *p* | *d* [95% CI] |
| within-ToM IRC Z ~ MASC (df=52) | | | | | | | | | |
| MASC Score  Group  MASC*group  mFD  Age  Sex  FSIQ  Site | 0.12  3.95  4.27  2.99  0.86  1.04  0.16  0.54 | 0.73  0.05  0.04  0.09  0.36  0.31  0.69  0.47 | 0.002  0.06  0.06  0.05  0.01  0.02  0.002  0.01 | -2.6e-03  -3.2e-01  1.10-02  -2.6e-01  -3.7e-03  -4.1e-02  1.6e-05  2.2e-02 | -1.81-01  -1.6e+00  1.9e+00  -2.12e-01  -2e-01  -1.9e-01  1.9e-03  1.1e-01 | 2.4e-03  1.9e-01  5.4e-03  1.5e-01  3 e-03  2.8e-02  1.2e-03  3.5e-02 | -1.09  -1.73  2.07  -1.71  -1.23  -1.44  0.01  0.63 | 0.28  0.09  0.04  0.09  0.23  0.16  0.99  0.53 | -0.13 [-0.61, 0.35]  -0.21 [-0.69, 0.27]  -0.21 [-0.69, 0.27]  -0.15 [-0.63, 0.33]  -0.18 [-0.66, 0.30]  0.002 [-0.48, 0.48]  0.08 [-0.40, 0.56]  0.25 [-0.23, 0.73] |
| within-ToM IRC Z ~ AQ (df=60) | | | | | | | | | |
| AQ Score  mFD  Age  Sex  FSIQ  Site | 1.29  2.37  1.41  0.20  1.34  0.45 | 0.26  0.13  0.24  0.66  0.25  0.50 | 0.02  0.04  0.02  0.003  0.02  0.01 | -0.002  -0.52  -0.004  -0.01  -0.001  0.02 | -0.17  -0.20  -0.21  -0.04  -0.10  0.11 | 0.001  0.34  0.003  0.03  0.001  0.03 | -1.35  -1.53  -1.40  -0.33  -0.77  0.67 | 0.18  0.13  0.17  0.74  0.44  0.50 | -0.16 [-0.64, 0.32]  -0.19 [-0.67, 0.29]  -0.17 [-0.65, 0.31]  -0.04 [-0.52, 0.44]  -0.09 [-0.57, 0.39]  0.08 [-0.40, 0.56] |
| T04 peak magnitude ~ MASC (df=53) | | | | | | | | | |
| MASC Score  mFD  Age  Sex  FSIQ  Site | 0.04  2.64  0.37  0.82  1.19  0.17 | 0.85  0.11  0.55  0.37  0.28  0.68 | 0.001  0.05  0.01  0.01  0.02  0.003 | -0.005  -0.91  -0.002  0.10  0.004  -0.06 | -0.08  -0.20  -0.02  0.12  0.14  -0.08 | 0.01  0.60  0.01  0.11  0.005  0.14 | -0.53  -1.50  -0.14  0.91  0.92  -0.42 | 0.60  0.14  0.89  0.37  0.36  0.68 | -0.06 [-0.54, 0.41]  -0.18 [-0.66, 0.30]  -0.02 [-0.50, 0.46]  0.11 [-0.37, 0.59]  0.11 [-0.37, 0.59]  -0.05 [-0.53, 0.43] |
| within-Pain IRC Z ~ EQ (df=59) | | | | | | | | | |
| EQ Score  mFD  Age  Sex  FSIQ  Site | 0.28  2.11  2.81  0.56  2.95  0.03 | 0.60  0.15  0.10  0.46  0.09  0.87 | 0.004  0.03  0.04  0.01  0.04  0.0004 | -0.0002  -0.28  -0.004  -0.02  -0.002  0.006 | -0.03  -0.22  -0.23  -0.08  -0.20  0.03 | 0.001  0.16  0.003  0.03  0.001  0.04 | -0.26  -1.75  -1.52  -0.68  -1.48  0.16 | 0.79  0.08  0.14  0.50  0.14  0.87 | -0.03 [-0.51, 0.45]  -0.21 [-0.69, 0.27]  -0.18 [-0.66, 0.30]  -0.08 [-0.56, 0.40]  -0.18 [-0.66, 0.30]  0.02 [-0.46, 0.50] |

*Abbreviations:* AQ, Autism Spectrum Quotient, b, unstandardized beta, B, standardized beta, CI, Confidence Interval, *d*, Cohen’s d, eta^2^, partial eta squared, EQ, Empathy Quotient, F, F value, FSIQ, Full Scale Intelligence Quotient, IRC, Inter-region correlations, MASC, Movie for the Assessment of Social Cognition, mFD, mean framewise displacement for all timepoints, *p,* p value, SE, standard error, t, t value, z, Fisher Z-transformed

**Table S9.** Participant characteristics - full sample before motion exclusion

|  | FULL (unmatched) SAMPLE (N = 116) | | | | |
| --- | --- | --- | --- | --- | --- |
|  | ASD  (N = 34) | NT  (N = 73) | chi^2^ / t | *p* | *V / d* |
| Sex (F/M) | 11/27 | 23/55 | 0.004 | 0.95 | 0.01 |
| Hand  (R/L/A) | 31/7/0 | 68/8/2 | 2.39 | 0.30 | 0.14 |
| Age  (years) | 18 - 46  27.1 (5.4) | 19 - 55  27.8 (7.3) | 1.03 | 0.31 | 0.04 |
| FSIQ | n=37 | n=73 |  | | |
|  | 84 - 142  112.7 (13.1) | 87 - 136  109.8 (10.4) | 1.13 | 0.26 | 0.26 |
| AQ | n=38 | n=78 |  | | |
|  | 10 - 48  27.5 (8.9) | 4 - 40  16.2 (6.7) | 7.6 | 1.19e-11 | 1.51 |
| MASC | n=32 | n=73 |  | | |
|  | 7 - 42  29.4 (8.3) | 27 - 43  35.6 (3.4) | 5.75 | 9.65e-08 | 1.16 |
| EQ | n=37 | n=78 |  | | |
|  | 9 - 90  32.2 (16.7) | 6 - 75  47.1 (13.9) | 5.63 | 1.37e-07 | 1.01 |
| exclTR | 0 - 176  63.7 (48.6) | 0 - 377  50.12 (72.3) | 1.05 | 0.30 | 0.21 |
| mFD | 0.06 - 0.45  0.2 (0.10) | 0.05 - 0.56  0.2 (0.1) | 1.54 | 0.13 | 0.30 |

Note: Values represent range, mean and standard deviation (SD); All t tests reflect group differences after controlling for group*site interactions.

*Abbreviations*: ASD, autism spectrum disorder; AQ, Autism Spectrum Quotient; d, Cohen’s d; EQ, Empathy Quotient; exclTR, number of excluded TR; F, female; FSIQ, Full Scale Intelligence Quotient; Hand, Handedness; M, male; MASC, Movie for the Assessment of Social Cognition; mFD, mean framewise displacement across all TR; n/N, number of subjects; NT, neurotypical; TR, repetition time

**Table S10.** Bayesian Linear Regression for Network connectivity (IRC) within the Pain network: Model Comparison (Null model vs. model comparing group) and posterior Summaries of Coefficients (Full Matched Sample).

|  | | | | | | | | | | | |
| --- | --- | --- | --- | --- | --- | --- | --- | --- | --- | --- | --- |
| **Models** | | **P(M)** | | **P(M\|data)** | | **BF_M_** | | **BF_10_** | | **R²** | |
| Null model (incl. mFD, Age, Site, Sex, FSIQ) |  | 0.500 |  | 0.684 |  | 2.169 |  | 1.000 |  | 0.086 |  |
| Group |  | 0.500 |  | 0.316 |  | 0.461 |  | 0.461 |  | 0.088 |  |
|  | | | | | | | | | | | |
| *Note.*  All models include mFD Age, Site, Sex, FSIQ. | | | | | | | | | | | |

|  | | | | | | | | | | | | | | | | | | | |
| --- | --- | --- | --- | --- | --- | --- | --- | --- | --- | --- | --- | --- | --- | --- | --- | --- | --- | --- | --- |
|  | | | | | | | | | | | | | | | | **95% Credible Interval** | | | |
| **Coefficient** | | **P(incl)** | | **P(excl)** | | **P(incl\|data)** | | **P(excl\|data)** | | **BF_inclusion_** | | **Mean** | | **SD** | | **Lower** | | **Upper** | |
| Intercept |  | 1.0 |  | 0.0 |  | 1.0 |  | 0.0 |  | 1.0 |  | 0.414 |  | 0.010 |  | 0.393 |  | 0.434 |  |
| mFD |  | 1.0 |  | 0.0 |  | 1.0 |  | 0.0 |  | 1.0 |  | -0.060 |  | 0.113 |  | -0.293 |  | 0.155 |  |
| Age |  | 1.0 |  | 0.0 |  | 1.0 |  | 0.0 |  | 1.0 |  | -0.003 |  | 0.002 |  | -0.006 |  | 1.366e-4 |  |
| Site |  | 1.0 |  | 0.0 |  | 1.0 |  | 0.0 |  | 1.0 |  | -0.006 |  | 0.024 |  | -0.057 |  | 0.040 |  |
| Sex |  | 1.0 |  | 0.0 |  | 1.0 |  | 0.0 |  | 1.0 |  | -0.019 |  | 0.020 |  | -0.062 |  | 0.018 |  |
| FSIQ |  | 1.0 |  | 0.0 |  | 1.0 |  | 0.0 |  | 1.0 |  | -0.001 |  | 8.638e-4 |  | -0.003 |  | 5.751e-4 |  |
| Group |  | 0.500 |  | 0.500 |  | 0.316 |  | 0.684 |  | 0.461 |  | -0.002 |  | 0.012 |  | -0.035 |  | 0.021 |  |
|  | | | | | | | | | | | | | | | | | | | |

*Abbreviations:* BF_M_, Bayes factor on the model odds; BF_10_ , Bayes Factor for relative predictive adequacy; excl, excluded; FSIQ, Full Scale Intelligence Quotient; incl, included; IRC, Inter-region correlations, mFD, mean framewise displacement for all timepoints, P, prior probability; SD, standard deviation

**REFERENCES**

Abraham, Alexandre, Fabian Pedregosa, Michael Eickenberg, Philippe Gervais, Andreas Mueller, Jean Kossaifi, Alexandre Gramfort, Bertrand Thirion, and Gael Varoquaux. 2014. “Machine Learning for Neuroimaging with Scikit-Learn.” Frontiers in Neuroinformatics 8. https://doi.org/10.3389/fninf.2014.00014.

Avants, B.B., C.L. Epstein, M. Grossman, and J.C. Gee. 2008. “Symmetric Diffeomorphic Image Registration with Cross-Correlation: Evaluating Automated Labeling of Elderly and Neurodegenerative Brain.” Medical Image Analysis 12 (1): 26–41. https://doi.org/10.1016/j.media.2007.06.004.

Behzadi, Yashar, Khaled Restom, Joy Liau, and Thomas T. Liu. 2007. “A Component Based Noise Correction Method (CompCor) for BOLD and Perfusion Based fMRI.” NeuroImage 37 (1): 90–101. https://doi.org/10.1016/j.neuroimage.2007.04.042.

Cox, Robert W., and James S. Hyde. 1997. “Software Tools for Analysis and Visualization of fMRI Data.” NMR in Biomedicine 10 (4-5): 171–78. https://doi.org/10.1002/(SICI)1099-1492(199706/08)10:4/5<171::AID-NBM453>3.0.CO;2-L.

Dale, Anders M., Bruce Fischl, and Martin I. Sereno. 1999. “Cortical Surface-Based Analysis: I. Segmentation and Surface Reconstruction.” NeuroImage 9 (2): 179–94. https://doi.org/10.1006/nimg.1998.0395.

Esteban, Oscar, Ross Blair, Christopher J. Markiewicz, Shoshana L. Berleant, Craig Moodie, Feilong Ma, Ayse Ilkay Isik, et al. 2018. “FMRIPrep.” Software. Zenodo. https://doi.org/10.5281/zenodo.852659.

Esteban, Oscar, Christopher Markiewicz, Ross W Blair, Craig Moodie, Ayse Ilkay Isik, Asier Erramuzpe Aliaga, James Kent, et al. 2018. “fMRIPrep: A Robust Preprocessing Pipeline for Functional MRI.” Nature Methods. https://doi.org/10.1038/s41592-018-0235-4.

Evans, AC, AL Janke, DL Collins, and S Baillet. 2012. “Brain Templates and Atlases.” NeuroImage 62 (2): 911–22. https://doi.org/10.1016/j.neuroimage.2012.01.024.

Fonov, VS, AC Evans, RC McKinstry, CR Almli, and DL Collins. 2009. “Unbiased Nonlinear Average Age-Appropriate Brain Templates from Birth to Adulthood.” NeuroImage 47, Supplement 1: S102. https://doi.org/10.1016/S1053-8119(09)70884-5.

Gorgolewski, K., C. D. Burns, C. Madison, D. Clark, Y. O. Halchenko, M. L. Waskom, and S. Ghosh. 2011. “Nipype: A Flexible, Lightweight and Extensible Neuroimaging Data Processing Framework in Python.” Frontiers in Neuroinformatics 5: 13. https://doi.org/10.3389/fninf.2011.00013.

Gorgolewski, Krzysztof J., Oscar Esteban, Christopher J. Markiewicz, Erik Ziegler, David Gage Ellis, Michael Philipp Notter, Dorota Jarecka, et al. 2018. “Nipype.” Software. Zenodo. https://doi.org/10.5281/zenodo.596855.

Greve, Douglas N, and Bruce Fischl. 2009. “Accurate and Robust Brain Image Alignment Using Boundary-Based Registration.” NeuroImage 48 (1): 63–72. https://doi.org/10.1016/j.neuroimage.2009.06.060.

Jenkinson, Mark, Peter Bannister, Michael Brady, and Stephen Smith. 2002. “Improved Optimization for the Robust and Accurate Linear Registration and Motion Correction of Brain Images.” NeuroImage 17 (2): 825–41. https://doi.org/10.1006/nimg.2002.1132.

Klein, Arno, Satrajit S. Ghosh, Forrest S. Bao, Joachim Giard, Yrjö Häme, Eliezer Stavsky, Noah Lee, et al. 2017. “Mindboggling Morphometry of Human Brains.” PLOS Computational Biology 13 (2): e1005350. https://doi.org/10.1371/journal.pcbi.1005350.

Lanczos, C. 1964. “Evaluation of Noisy Data.” Journal of the Society for Industrial and Applied Mathematics Series B Numerical Analysis 1 (1): 76–85. https://doi.org/10.1137/0701007.

Power, Jonathan D., Anish Mitra, Timothy O. Laumann, Abraham Z. Snyder, Bradley L. Schlaggar, and Steven E. Petersen. 2014. “Methods to Detect, Characterize, and Remove Motion Artifact in Resting State fMRI.” NeuroImage 84 (Supplement C): 320–41. https://doi.org/10.1016/j.neuroimage.2013.08.048.

Satterthwaite, Theodore D., Mark A. Elliott, Raphael T. Gerraty, Kosha Ruparel, James Loughead, Monica E. Calkins, Simon B. Eickhoff, et al. 2013. “An improved framework for confound regression and filtering for control of motion artifact in the preprocessing of resting-state functional connectivity data.” NeuroImage 64 (1): 240–56. https://doi.org/10.1016/j.neuroimage.2012.08.052.

Tustison, N. J., B. B. Avants, P. A. Cook, Y. Zheng, A. Egan, P. A. Yushkevich, and J. C. Gee. 2010. “N4ITK: Improved N3 Bias Correction.” IEEE Transactions on Medical Imaging 29 (6): 1310–20. https://doi.org/10.1109/TMI.2010.2046908.

Zhang, Y., M. Brady, and S. Smith. 2001. “Segmentation of Brain MR Images Through a Hidden Markov Random Field Model and the Expectation-Maximization Algorithm.” IEEE Transactions on Medical Imaging 20 (1): 45–57. https://doi.org/10.1109/42.906424.
